# Supplementary material for: Chromoselective Photocatalysis Enables Stereocomplementary Biocatalytic Pathways
Source: Angew Chem Int Ed Engl. 2021 Feb 26;60(13):6965–9. doi: 10.1002/anie.202100164 (PMC8048449; doi:10.1002/anie.202100164)
Supplement: Supplementary file 1 — Supplementary [file ANIE-60-6965-s001.pdf]

Angewandte Chemie
Eine Zeitschrift der Gesellschaft Deutscher Chemiker

Supporting Information

Chromoselective Photocatalysis Enables Stereocomplementary Biocatalytic Pathways**

Luca Schmermund, Susanne Reischauer, Sarah Bierbaumer, Christoph K. Winkler, Alba Diaz-Rodriguez, Lee J. Edwards, Selin Kara, Tamara Mielke, Jared Cartwright, Gideon Grogan, Bartholomäus Pieber, and Wolfgang Kroutil**

anie_202100164_sm_miscellaneous_information.pdf

Table of Contents

SUPPORTING INFORMATION 1

General Information 4

Chemicals 4
Thin Layer Chromatography and Flash Column Chromatography 4
Spectroscopy 4
Gas Chromatography 4
Optical Rotation 5

Photoreactor Set Up 5

Photoreactor Set Up for Initial Studies of the Oxidation of Ethylbenzene using CN-OA-m 5
Photoreactor for Photo-Chemo-Enzymatic Reactions in Glass Vials 6
GSK Photoreactor for Photo-Chemo-Enzymatic Reactions in Microwave Tubes 7

Expression of *Aae*UPO and ADH-A 8

Expression of *Aae*UPO 8
Expression and Purification of ADH-A 8

Photocatalytic Oxidation of Ethylbenzene and *rac*-1-phenylethanol using CN-OA-m with Different Wavelengths 10

ADH-A Catalysed Reduction of Acetophenone under the Presence of Hydrogen Peroxide 11

Experimental Procedures 12

Experimental Procedure for the Synthesis of Racemic Reference Compounds 12

rac-1-(3,5-difluorophenyl)ethan-1-ol 12
rac-1-(2-bromophenyl)ethan-1-ol 12
rac-1-(4-(1-hydroxyethyl)phenyl)ethan-1-one 13

Experimental Procedure for Photo-Chemo-Enzymatic Hydroxylations 13
General Procedure for Photo-Chemo-Enzymatic Hydroxylations with CN-OA-m and UPO in Glass Vials 13

(*R*)-1-phenylethan-1-ol 13
(*R*)-1-(*o*-tolyl)ethan-1-ol 14
(*R*)-1-(*m*-tolyl)ethan-1-ol 14
(*R*)-1-(*p*-tolyl)ethan-1-ol 14
2-phenylpropan-2-ol 14
(*R*)-1-(3,5-difluorophenyl)ethan-1-ol 15
(*R*)-1-(4-bromophenyl)ethan-1-ol 15
(*R*)-1-(2-bromophenyl)ethan-1-ol 15
(*R*)-1-(4-(1-hydroxyethyl)phenyl)ethan-1-one 15
(*R*)-1-(4-(1-hydroxyethyl)phenyl)propan-1-one 16

General Procedure for Photo-Chemo-Enzymatic Cascade Reactions with CN-OA-m and ADH. 16
Photocatalytic Oxidation of Ethylbenzene with CN-OA-m in the Photoreactor for Photo-Chemo-Enzymatic Reactions 16
ADH-A Catalysed Reduction of Acetophenone in the Presence of Hydrogen Peroxide 17

GC Analytics 18

Additional Experiments 33

Influence of the MeOH Concentration 33
Influence of the Enzyme Concentration 33
Influence of the Amount of CN-OA-m 34
Influence of the Light Intensity 34
*Aae*UPO Stability under Different Wavelengths 35
Reuse of CN-OA-m 35
Comparison of CN-OA-m before and after the Photo-Chemo-Enzymatic Reaction with AaeUPO 36
DNA and Protein Sequences 37
Abbreviations 38
Literature 39

General Information

Chemicals

General reagents, substrates and solvents were purchased and used as supplied from Sigma-Aldrich (Merck KGaA), TCI, Alfa Aesar, Thermo Fisher Scientific, AmBeed, Enamine and Lancaster.
The CN-OA-m photocatalyst was produced by co-condensation of urea and oxamide followed post-calcination in a molten salt according to a literature procedure.[1]

Thin Layer Chromatography and Flash Column Chromatography

For the **thin layer chromatography (TLC)** Merck TLC silica gel 60 F_{254} plates were used. The compounds were visualized by using UV-light (254 nm/366 nm) or basic aqueous potassium permanganate stain (2.5 g $NaHCO_3$, 0.67 g $KMnO_4$, 0.2 mL acetic acid, 200 mL), followed by heating with a heat gun.

Flash column chromatography was used to purify crude products. The purification was performed using silica gel 60 M (particle size 40-63 µm/ 230-400 mesh) from Merck as stationary phase under excess pressure.

Spectroscopy

^{1}H-NMR spectra were recorded on AV II 300 MHz spectrometer from Bruker Physics in chloroform-*d_1* ($CDCl_3$). The chemical shift δ is indicated in parts per million (ppm) relative to the internal standard trimethylsilane (δ = 0 ppm). The spectra were calibrated using the residual proton signal of the solvent $CDCl_3$ at 7.26 ppm internal references.[2] The coupling constants (J) are given in Hz. The following abbreviations were used to designate multiplicities in the recorded spectra: s (singlet), d (doublet), t (triplett), q (quartet), quint (quintet) and m (multiplet).

^{13}C-NMR spectra were recorded on AV-300 (75.5 MHz) spectrometer from Bruker Physics in $CDCl_3$. The chemical shift δ is indicated in parts per million (ppm) relative to the internal standard trimethylsilane (δ = 0 ppm). The spectra were calibrated using the carbon signal of the solvent $CDCl_3$ at 77.16 ppm.[2]

Gas Chromatography

For **gas chromatography (GC)** an Agilent GC7890A system with FID-detector (heater 300 °C, H_2-flow 28 mL/min, Air-flow 350 mL/min, makeup flow 25 mL/min; Date Rate/min peak width 50 Hz/0.004 min) was

used. For general measurements a 30 m CP WAX 52CB column (Varian) with 0.25 µm inner diameter was used. Heater was at 250 °C, 0.60 bar, gas saver 15 mL/min after 2 min. For determination of optical purities, a 25 m CP-ChiraSil-DEX CB column (Agilent Technologies) with 0.0.32 µm inner diameter was used. Heater was at 220 °C, 0.17 bar, gas saver 15 mL/min after 2 min.

Optical Rotation

Optical rotation values were measured on a Perkin Elmer Polarimeter 341.

Photoreactor Set Up

For the photocatalytic and photochemoenzymatic reactions different photoreactor systems were used:

Photoreactor Set Up for Initial Studies of the Oxidation of Ethylbenzene using CN-OA-m

Experiments using blue light were carried out using a Kessil PR160-440 LED (Fig. S1). Two sealed reaction vessels were placed on a stirring plate 4.5 cm away from a single lamp. To avoid heating of the reaction mixture, a fan was used for cooling. All reactions were performed with maximum stirring speed.

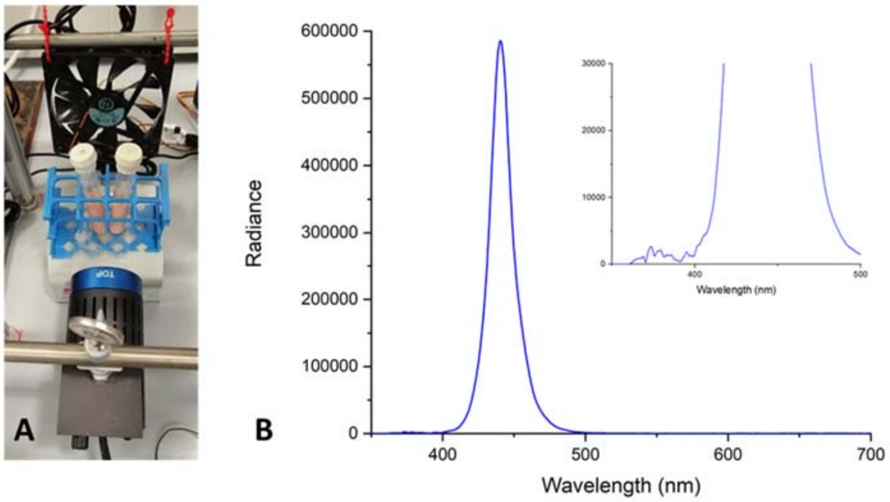

Figure S1. Setup for blue light experiments (A); Emission spectra of the Kessil PR160-440 (B).

Experiments using green light were carried out using a Kessil PR160-525 LED (Fig. S2). Two sealed reaction vessels were placed on a stirring plate 4.5 cm away from a single lamp. To avoid heating of the reaction mixture, a fan was used for cooling. All reactions were performed with maximum stirring speed.

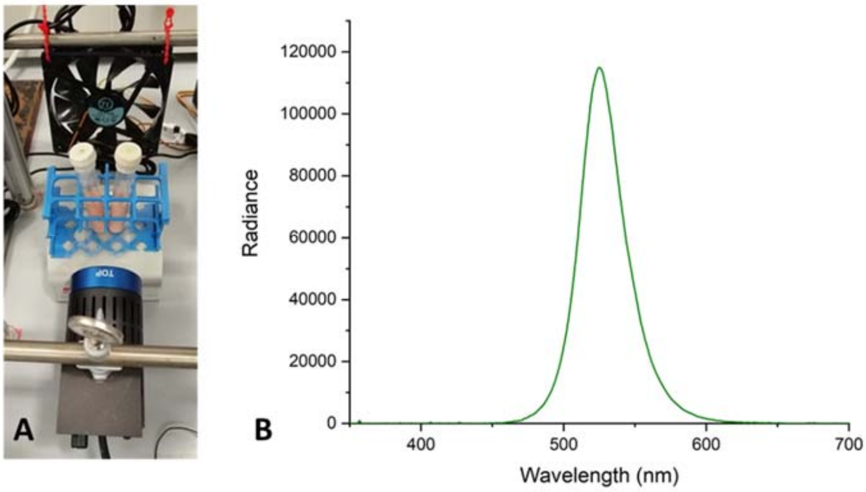

Figure S2. Setup for green light experiments (A). Emission spectra of the Kessil PR160-525 (B).

Photoreactor for Photo-Chemo-Enzymatic Reactions in Glass Vials

For reactions performed in crimp glass vials (1.5 mL, Merck, Fig. S3) a self-built photoreactor (Fig. S4) was used. The glass vials were closed with crimp seals (Merck) and placed in the photoreactor for irradiation.

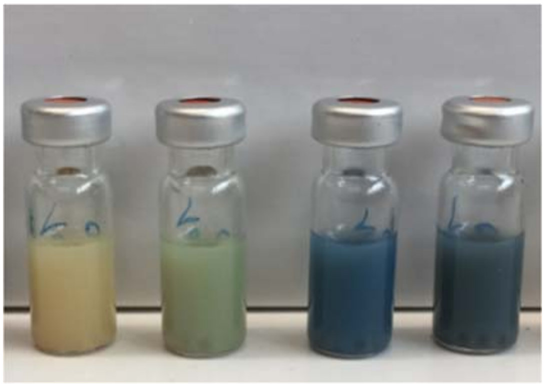

Figure S3. Crimp glass vial (1.5 mL) used for biotransformations with CN-OA-m and UPO (total volume 1 mL).

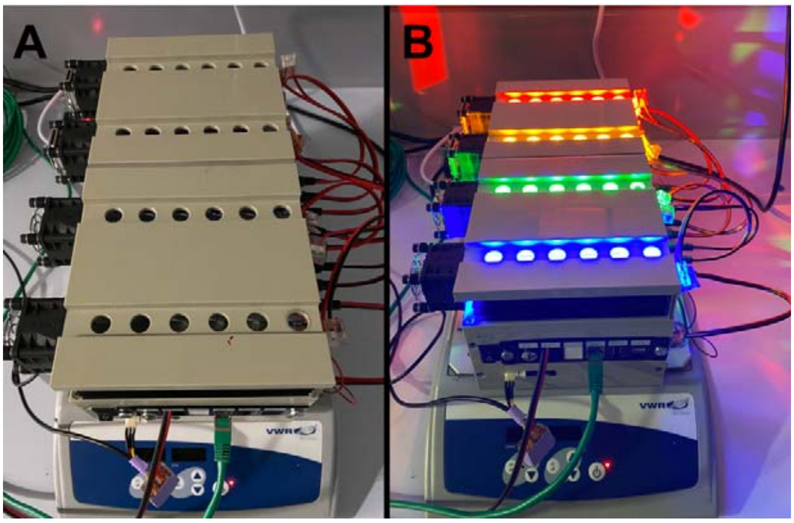

Figure S4. Photoreactor used for the CN-OA-m/UPO system; A) Photoreactor off; B) Photoreactor on: The reactor has four rows with six reactions slots (24 in total) for 1.5 mL glass vials. Every row has two LED stripes (Lumitronix, PowerBar V3 LED-Modul Aluminium 12x Osram Oslon SSL LEDs) and every glass vial is irradiated by two LED lamps. The LED stripes are interchangeable allowing to perform reactions at different wavelengths (white light >450 nm, 405 nm, 455 nm, 470 nm, 528 nm, 590 nm). Temperature and light intensity are independently adjustable for each LED row. Shaking was performed at 500 rpm.
The emission spectra and specification sheets of the LED lamps are given by the supplier.

GSK Photoreactor for Photo-Chemo-Enzymatic Reactions in Microwave Tubes

For reactions performed in microwave reaction vials (Biotage, Microwave Reaction Kit, 2-10 mL) a photoreactor[3] provided by GSK (electronics designed by Pacer Components Ltd. Pangbourne, UK; housing machined by Rosper Engineering Co., Harlow, UK) was used (Fig. S5). A stirring bar was added, the microwave reaction vials were closed with crimp seals and placed in the photoreactor for irradiation. The emission spectra of the LED stripes used for photochemoenzymatic reactions and the characterization of the photoreactor can be found in the provided literature.[3]

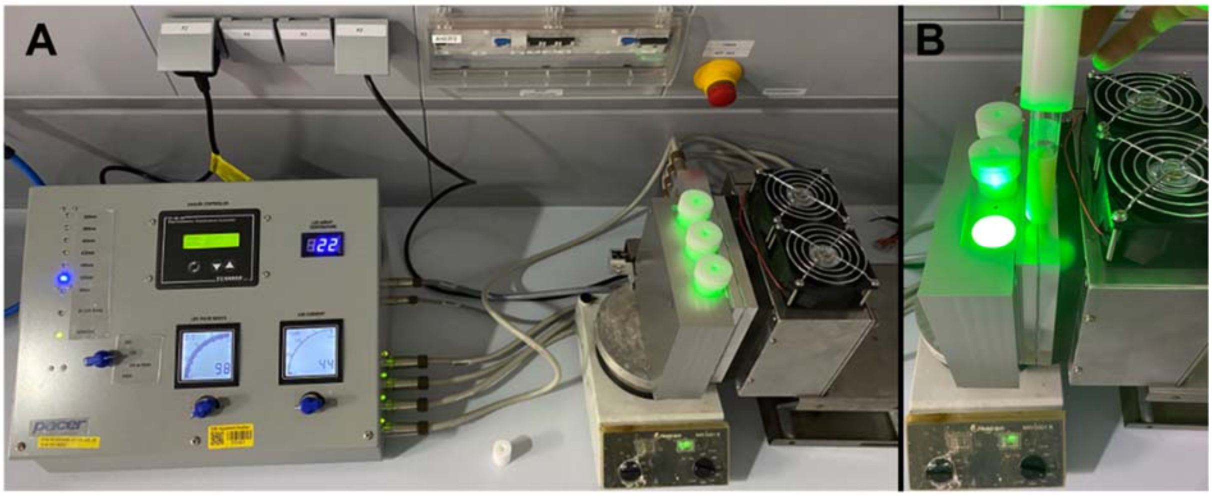

Figure S5. Photoreactor used for the CN-OA-m/UPO system; A) Complete photoreactor unit with control panel unit (left) and reactor and cooling unit (right); B) Reactor unit on a magnetic stirrer with microwave reaction vials: The reactor has three slots for microwave reaction vials. A magnetic stirrer is used to stir the reaction (500 rpm). The photoreactor is controlled by a control unit allowing to adjust the temperature, light intensity (25-500 mA), and wavelength. Six different wavelengths can be used for reactions (365 nm, 385 nm, 405 nm, 420 nm, 455 nm and 528 nm).

Expression of *Aae*UPO and ADH-A

Expression of *Aae*UPO

Expression, isolation and lyophilisation of *Aae*UPO were performed as described in Reference [3].

Expression and Purification of ADH-A

For the expression of ADH-A (ADH-A-pET21a-strep, internal number pEG518), LB-medium (700 mL) containing the ampicillin (100 µg/mL) was inoculated in a 2 L baffled flask with 1% *Escherichia coli* BL21(DE3)/pEG518. The culture was incubated at 30 °C and 120 rpm until an OD_{600} of 5 was reached. Then induction was performed by adding IPTG (2 mM). Further incubation took place at 20 °C and 120 rpm for 24 h. Afterwards, the cell suspension was centrifuged (5000 rpm, 20 min, 4 °C), the supernatant discarded, and the pellets were resuspended in Tris buffer (15 mL, 50 mM TRIS/HCl, pH 7.5). The suspension was centrifuged (8 °C, 4500 rpm, 20 min), the supernatant was discarded and the pellet either lysed (50 mM Tris/HCl, 150 mM NaCl, 20 mM imidazole, pH 7.5) or shock frozen in liquid nitrogen and stored at -20 °C till further use.

To purify the protein the harvested cells were resuspended in lysis buffer (10 mL/g pellet, 50 mM Tris/HCl, 150 mM NaCl, 20 mM imidazole, pH 7.5) and lysed on ice by sonication (3x, 2 min 30 sec, 30% amplitude, 2.0 sec pulse on, 4.0 sec pulse off, 1 min pause, Digital sonifier, Branson). The cell suspension was centrifuged (20 min, 18000 rpm, 4 °C) and the clear slightly yellow cell free extract (CFE) was filtered (0.45 µm syringe filter) and stored on ice for protein purification.

The ADH-A bearing a strep-Tag were purified by strep-tactin affinity chromatography (Strep-Tactin®XT Superflow® Column, IBA) with gravity flow.

The purification was performed at 4 °C. The column was equilibrated with 2 CV buffer W (100 mM Tris/HCl, 150 mM NaCl, pH 8) before the filtered CFE was loaded onto the column and the flow through collected for SDS-PAGE-sample. The column was then 5x washed with 1 CV buffer W. Then, the protein was eluted by applying 8x 0.5 CV Buffer BXT (100 mM Tris/HCl, 150 mM NaCl, 50 mM biotin, pH 8). After finished elution the column was regenerated with 4 CV 10 mM sodium hydroxide (NaOH). NaOH was removed immediately by washing the column 2x with 4 CV Buffer W. The column was stored at 4 °C.

To exchange the buffer a Sephadex G-25 PD10 desalting column (GE Healthcare) was equilibrated with KP_i buffer. The concentrated protein solution (2.5 mL) was loaded onto the column and eluted with KP_i buffer (exactly 3.5 mL). The final enzyme solution was aliquoted and stored at -20 °C.

Photocatalytic Oxidation of Ethylbenzene and *rac*-1-phenylethanol using CN-OA-m with Different Wavelengths

Table S1. Photocatalytic reaction of ethylbenzene in the presence of CN-OA-m using the setups described in Figure S1 and S2.

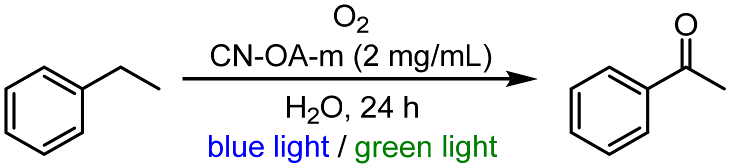

Entry[a]	additive	light	Ratio ethylbenzene: acetophenone[b]
1	MeOH	blue	1:1
2	MeOH	green	1:0
3	-	blue	0:1
4	-	green	1:0

[a] Reaction conditions: ethylbenzene (180 µmol), 12 mg CN-OA-m in water (3 mL) or water and MeOH (1:1), 440 nm blue LED or 525 nm green LED (50% power) at 21 °C for 24 h; [b] Determined by ^{1}H-NMR after extraction of the entire reaction mixture with $CDCl_3$.

Table S2. Photocatalytic reaction of *rac*-1-phenylethanol in the presence of CN-OA-m using the setups described in Figure S1 and S2.

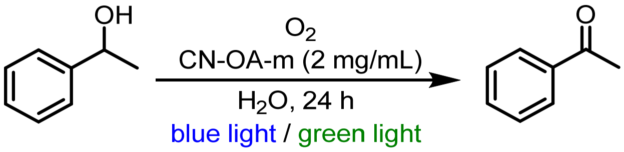

Entry	additive	light	Ratio *rac*-1-phenylethanol: acetophenone[b]
1	MeOH	blue	1:0.19
2	MeOH	green	1:traces
3	-	blue	1:1.8
4	-	green	1:0.05

[a] Reaction conditions: *rac*-1-phenylethanol (180 µmol), 12 mg CN-OA-m in water (3 mL) or water and MeOH (1:1), 440 nm blue LED or 525 nm green LED (50% power) at 21 °C for 24 h. [b] Determined by ^{1}H-NMR after extraction of the entire reaction mixture with $CDCl_3$.

Table S3. Photocatalytic reaction of ethylbenzene in the presence of CN-OA-m in the photoreactor used for photo-chemo-enzymatic reactions, which is described in Figure S4.

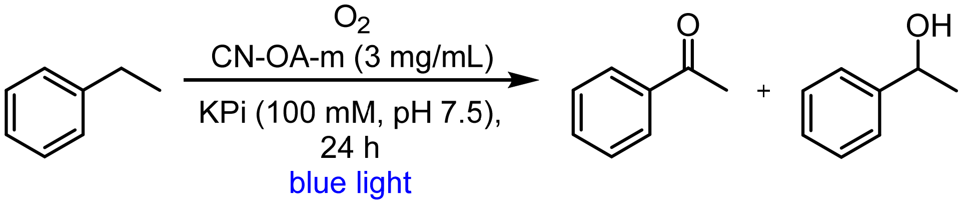

Entry[a]	additive	light	acetophenone[b]	1-phenylethanol[b]
1	MeOH	blue	2.7 mM	0.3 mM

[a] Reaction conditions: ethylbenzene (10 mM), CN-OA-m (3 mg/mL), MeOH (1% v/v), KPi buffer (100 mM, pH 7.5), LED 455 nm, 1998 µmol photons m^{-2} s^{-1}, 30 °C, 24 h. [b] GC yield.

ADH-A Catalysed Reduction of Acetophenone under the Presence of Hydrogen Peroxide

Table S4. ADH-A catalysed reduction of acetophenone in the presence of hydrogen peroxide.

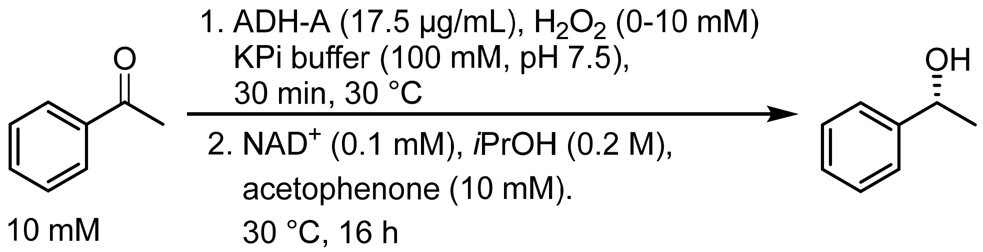

Entry[a]	c_{H2O2}	$c_{1\text{-phenylethanol}}$[b]	*e.e.*[c]
1	10	8.9 mM	>99% (*S*)
2	2	8.7 mM	>99% (*S*)
3	0	9.0 mM	>99% (*S*)

[a] Reaction conditions: 1) H_2O_2 (0, 2, 10 mM), ADH-A (17.5 µg/mL), KPi buffer (100 mM, pH 7.5), 30 °C, 30 min; 2) acetophenone (10 mM), NAD^+ (0.1 mM), *i*PrOH (0.2 M), KPi buffer (100 mM, pH 7.5), 30 °C, 16 h; [b] GC yield; [c] Determined by GC.

Experimental Procedures

Experimental Procedure for the Synthesis of Racemic Reference Compounds

The respective ketone (1.0 eq) was dissolved in EtOH (10 mL). Then the solution was cooled to 0 °C with an ice water bath and sodium borohydride (1.5 eq) was added in one portion. The reaction was stirred for 10 min at 0 °C. Afterwards, the ice water bath was removed, and the reaction was stirred for 50 min at rt (the product formation was monitored by TLC). After complete consumption of the starting material the reaction was cooled to 0 °C with an ice water bath. Then water (3 mL) was added dropwise over 3 min followed by a dropwise addition of hydrochloric acid (37 wt.%, 3 mL) over 3 min. The ice water bath was removed, the reaction was stirred for 10 min at 21 °C and brine (10 mL) was added. The reaction was extracted with EtOAc (3x10 mL), the combined organic phases were dried over Na_2SO_4 and the solvent was removed under reduced pressure. The obtained crude product was purified by flash column chromatography.

rac-1-(3,5-difluorophenyl)ethan-1-ol

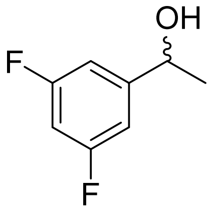

4

Batch: 1-ethyl-3,5-difluorobenzene (104 mg, 0.67 mmol, 1.0 eq) and sodium borohydride (37.8 mg, 1.0 mmol, 1.5 eq). The obtained crude product was purified via flash column chromatography (cyclohexane/EtOAc 8/1 to 4/1) to give **4** as a colourless oil (98.0 mg, 0.62 mmol, 93%).

R_f = 0.30 (cyclohexane/EtOAc 4/1); **^{1}H NMR** (300 MHz, $CDCl_3$) δ 6.9 (qt, *J* = 6.7, 2.2 Hz, 2H), 6.69 (tt, *J* = 8.9, 2.3 Hz, 1H), 4.88 (q, *J* = 6.5 Hz, 1H), 1.47 (d, *J* = 6.5 Hz, 3H) ppm; **^{13}C NMR** (75 MHz, $CDCl_3$) δ 164.9 (d, *J* = 12.6 Hz), 161.6 (d, *J* = 12.6 Hz), 150.1 (t, *J* = 8.2 Hz), 108.3 (d, *J* = 25.1 Hz), 108.3 (d, *J* = 9.3 Hz), 102.7 (t, *J* = 25.4 Hz), 69.6, 25.4 ppm.[4]

rac-1-(2-bromophenyl)ethan-1-ol

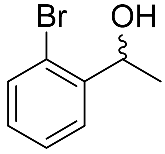

5

Batch: 1-bromo-2-ethylbenzene (162 mg, 0.82 mmol, 1.0 eq) and sodium borohydride (46.3 mg, 1.22 mmol, 1.5 eq). The obtained crude product was purified via flash column chromatography (cyclohexane/EtOAc 8/1 to 4/1) to give **5** as a colourless oil (153 mg, 0.77 mmol, 94%).

R_f = 0.13 (cyclohexane/EtOAc 4/1); **^{1}H NMR** (300 MHz, $CDCl_3$) δ 7.59 (dd, *J* = 7.8, 1.5 Hz, 1H), 7.51 (d, *J* = 8.0 Hz, 1H), 7.34 (t, *J* = 7.5 Hz, 1H), 7.12 (td, *J* = 7.7, 1.6 Hz, 1H), 5.24 (q, *J* = 6.4 Hz, 1H), 1.48 (d, *J* = 6.4 Hz, 3H) ppm; **^{13}C NMR** (75 MHz, $CDCl_3$) δ 144.7, 132.8, 128.8, 128.0, 126.8, 121.9, 69.3, 23.7 ppm.[5]

rac-1-(4-(1-hydroxyethyl)phenyl)ethan-1-one

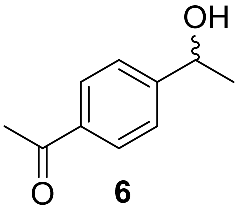
6

Batch: 1-(4-ethylphenyl)ethan-1-one (200 mg, 1.23 mmol, 1.0 eq) and sodium borohydride (23.3 mg, 0.62 mmol, 0.5 eq). The obtained crude product was purified via flash column chromatography (cyclohexane/EtOAc 7/1 to 3/1) to give **6** as a colourless oil (90.0 mg, 0.0.55 mmol, 89%).

R_f = 0.30 (cyclohexane/EtOAc 4/1); **^{1}H NMR** (300 MHz, $CDCl_3$) δ 7.91 (d, *J* = 8.3 Hz, 2H), 7.44 (d, *J* = 8.3 Hz, 2H), 4.95 (q, *J* = 6.5 Hz, 1H), 2.57 (s, 3H), 1.49 (d, *J* = 6.5 Hz, 3H) ppm;[6] **^{13}C NMR** (75 MHz, $CDCl_3$) δ 198.1, 151.4, 136.3, 128.7, 125.6, 77.2, 26.8, 25.4 ppm.

Experimental Procedure for Photo-Chemo-Enzymatic Hydroxylations

General Procedure for Photo-Chemo-Enzymatic Hydroxylations with CN-OA-m and UPO in Glass Vials

A crimp glass vial (1.5 mL) was charged with CN-OA-m (2.0 mg, 2.0 mg/mL) and five glass beads (1.0 mm, Merck). Then tricine buffer (840 µL, 100 mM, pH 7.5) was added and the mixture was sonicated in an ultrasonic bath (5 s) to ensure a fine distribution of the CN-OA-m. Then the substrate (10 mM, 10 µmol) dissolved in MeOH (1 M stock solution, 10.0 µL were used) and the *Aae*UPO (150 µL, 2.2 U/mg, 25 nM) from a stock solution (6 mg/mL *Aae*UPO in tricine buffer 6 mg/mL) were added. The crimp vial was sealed, and the glass vial was irradiated in the photoreactor (528 nm, 1330 µmol photons m^{-2} s^{-1}) for 24 h at 30 °C and 500 rpm. Afterwards, the reaction was extracted with EtOAc (1x 400 µL and 1x 400µL + 10 mM 1-octanol) and the combined organic phases were dried over Na_2SO_4. The extracts were analyzed by GC. For isolation and purification of the product 10-20 glass vial reactions were combined. The combined reactions were extracted with EtOAc (2x12 mL), the combined organic phase was dried over Na_2SO_4 and the solvent was removed under reduced pressure. The obtained crude product was purified via flash column chromatography.

(*R*)-1-phenylethan-1-ol

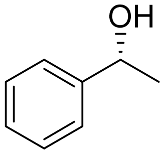
(*R*)-2a

Substrate: ethylbenzene (10 mM, 10 µM). The obtained crude product was purified via flash column chromatography (cyclohexane/EtOAc 7/1 to 4/1) to give **(*R*)-2a** as a colourless oil (9.3 mg, 78.0 µmol, 25%).

R_f = 0.40 (cyclohexane/EtOAc 4/1); **^{1}H NMR** (300 MHz, $CDCl_3$) δ 7.42–7.24 (m, 5H), 4.90 (q, *J* = 6.5 Hz, 1H), 1.50 (d, *J* = 6.5 Hz, 3H) ppm,[7] ***e.e.***= 99%.

(*R*)-1-(*o*-tolyl)ethan-1-ol

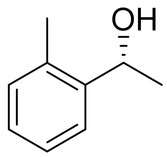

Substrate: 1-ethyl-2-methylbenzene (10 mM, 10 µM). The obtained crude product was purified via flash column chromatography (cyclohexane/EtOAc 7/1 to 4/1) to give **(*R*)-2b** as a colourless oil (10.2 mg, 75.0 µmol, 36%).

(*R*)-2b

R_f = 0.32 (cyclohexane/EtOAc 4/1); **^{1}H NMR** (300 MHz, $CDCl_3$) δ 7.56–7.49 (m, 1H), 7.29–7.11 (m, 3H), 5.14 (q, *J* = 6.4 Hz, 1H), 2.35 (s, 3H), 1.47 (d, *J* = 6.4 Hz, 3H) ppm,[8] ***e.e.***= 98%.

(*R*)-1-(*m*-tolyl)ethan-1-ol

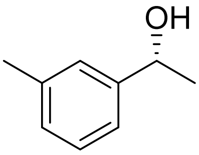

Substrate: 1-ethyl-3-methylbenzene (10 mM, 10 µM). The obtained crude product was purified via flash column chromatography (cyclohexane/EtOAc 7/1 to 4/1) to give **(*R*)-2c** as a colourless oil (1.6 mg, 11.7 µmol, 4.9%).

(*R*)-2c

R_f = 0.35 (cyclohexane/EtOAc 4/1); **^{1}H NMR** (300 MHz, $CDCl_3$) δ 7.28–7.14 (m, 1H), 7.09 (d, *J* = 7.3 Hz, 3H), 4.87 (q, *J* = 6.5 Hz, 3H), 2.36 (s, 9H), 1.49 (d, *J* = 6.5 Hz, 9H) ppm,[7b] ***e.e.***= 98%.

(*R*)-1-(*p*-tolyl)ethan-1-ol

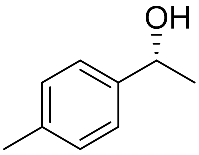

Substrate: 1-ethyl-4-methylbenzene (10 mM, 10 µM). The obtained crude product was purified via flash column chromatography (cyclohexane/EtOAc 7/1 to 4/1) to give **(*R*)-2d** as a colourless oil (2.1 mg, 17.5 µmol, 8.3%).

(*R*)-2d

R_f = 0.35 (cyclohexane/EtOAc 4/1); **^{1}H NMR** (300 MHz, $CDCl_3$) δ 7.29 (s, 5H), 7.16 (d, *J* = 7.9 Hz, 2H), 4.88 (q, *J* = 6.4 Hz, 1H), 2.35 (s, 3H), 1.49 (d, *J* = 6.4 Hz, 3H) ppm,[7] ***e.e.***= 98%.

2-phenylpropan-2-ol

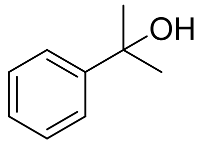

Substrate: cumene (10 mM, 10 µM). The obtained crude product was purified via flash column chromatography (cyclohexane/EtOAc 7/1 to 4/1) to give **(*R*)-2e** as a colourless oil (7.0 mg, 51.0 µmol, 25.0%).

2e

R_f = 0.34 (cyclohexane/EtOAc 5/1); **^{1}H NMR** (300 MHz, $CDCl_3$) δ δ 7.53–7.47 (m, 1H), 7.35 (t, *J* = 7.5 Hz, 1H), 7.28–7.21 (m, 1H), 1.59 (s, 3H) ppm.[9]

(*R*)-1-(3,5-difluorophenyl)ethan-1-ol

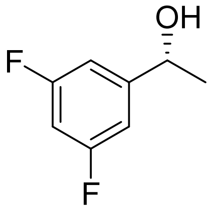

(*R*)-2f

Substrate: 1-ethyl-3,5-difluorobenzene (10 mM, 10 μM). The obtained crude product was purified via flash column chromatography (cyclohexane/EtOAc 7/1 to 4/1) to give **(*R*)-2f** as a colourless oil (10.1 mg, 64.0 μmol, 30%).

R_f = 0.26 (cyclohexane/EtOAc 5/1); **^{1}H NMR** (300 MHz, $CDCl_3$) δ 6.93–6.83 (m, 2H), 6.73–6.66 (m, 1H), 4.88 (q, *J* = 6.5 Hz, 1H), 1.48 (d, *J* = 6.5 Hz, 3H) ppm, ***e.e.***= 98%.

(*R*)-1-(4-bromophenyl)ethan-1-ol

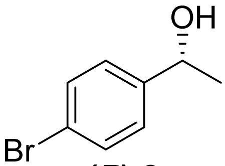

(*R*)-2g

Substrate: 1-bromo-4-ethylbenzene (10 mM, 10 μM). The obtained crude product was purified via flash column chromatography (cyclohexane/EtOAc 7/1 to 4/1) to give **(*R*)-2g** as a colourless oil (5.1 mg, 25.4 μmol, 11%).

R_f = 0.32 (cyclohexane/EtOAc 4/1); **^{1}H NMR** (300 MHz, $CDCl_3$) δ 7.47 (d, *J* = 8.4 Hz, 2H), 7.24 (d, *J* = 6.7 Hz, 2H), 4.86 (q, *J* = 6.4 Hz, 1H), 1.47 (d, *J* = 6.5 Hz, 3H) ppm,[5] ***e.e.***= 98%.

(*R*)-1-(2-bromophenyl)ethan-1-ol

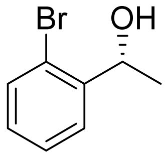

(*R*)-2h

Substrate: 1-bromo-2-ethylbenzene (10 mM, 10 μM). The obtained crude product was purified via flash column chromatography (cyclohexane/EtOAc 7/1 to 4/1) to give **(*R*)-2h** as a colourless oil (2.1 mg, 10.0 μmol, 5.0%).

R_f = 0.46 (cyclohexane/EtOAc 4/1); **^{1}H NMR** (300 MHz, $CDCl_3$) δ 7.60 (dd, *J* = 7.8, 1.5 Hz, 1H), 7.51 (d, *J* = 8.0 Hz, 1H), 7.35 (t, *J* = 7.2 Hz, 1H), 7.13 (td, *J* = 7.7, 1.6 Hz, 1H), 5.25 (q, *J* = 6.4 Hz, 1H), 1.49 (d, *J* = 6.4 Hz, 3H) ppm,[5] ***e.e.***= 98%.

(*R*)-1-(4-(1-hydroxyethyl)phenyl)ethan-1-one

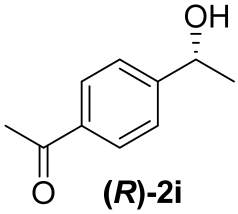

(*R*)-2i

Substrate: 1-(4-ethylphenyl)ethan-1-one (10 mM, 10 μM). The obtained crude product was purified via flash column chromatography (cyclohexane/EtOAc 6/1 to 4/1) to give **2i** as a colourless solid (10.5 mg, 64.0 μmol, 30.5%).

R_f = 0.13 (cyclohexane/EtOAc 4/1); **^{1}H NMR** (300 MHz, $CDCl_3$) δ 7.93 (d, *J* = 8.2 Hz, 2H), 7.46 (d, *J* = 8.3 Hz, 2H), 4.96 (q, *J* = 6.5 Hz, 1H), 2.59 (s, 3H), 1.50 (d, *J* = 6.5 Hz, 3H) ppm,[6] **optical rotation:** $[\alpha]_D^{20}$ = +49.7 ($CHCl_3$, c = 1.00), lit.: $[\alpha]_D^{20}$ = +41.7 ($CHCl_3$, c = 0.69);[10] $[\alpha]_D^{20}$ = -42.6 ($CHCl_3$, c = 1.00, *S*-enantiomer);[11] ***e.e.***= 99%.

(*R*)-1-(4-(1-hydroxyethyl)phenyl)propan-1-one

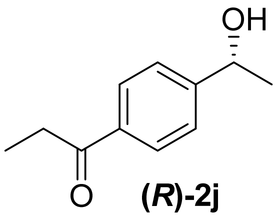

(*R*)-2j

Substrate: 1-(4-ethylphenyl)propan-1-one (10 mM, 10 µM). The obtained crude product was purified via flash column chromatography (cyclohexane/EtOAc 6/1 to 3/1) to give **(*R*)-2j** as a colourless solid (9.80 mg, 54.9 µmol, 26.1%).

R_f = 0.3 (cyclohexane/EtOAc 4/1); **^{1}H NMR** (300 MHz, $CDCl_3$) δ 7.94 (d, *J* = 8.3 Hz, 2H), 7.46 (d, *J* = 8.3 Hz, 2H), 4.96 (q, *J* = 6.5 Hz, 1H), 2.99 (q, *J* = 7.2 Hz, 2H), 1.50 (d, *J* = 6.5 Hz, 3H), 1.22 (t, *J* = 7.3 Hz, 3H) ppm; **optical rotation:** $[\alpha]_D^{20}$ = +41.9 ($CHCl_3$, c = 0.92), ***e.e.***= 98%.

General Procedure for Photo-Chemo-Enzymatic Cascade Reactions with CN-OA-m and ADH.

A crimp glass vial (1.5 mL) was charged with CN-OA-m (3.0 mg, 3.0 mg/mL) and five glass beads (1.0 mm, Merck). Then phosphate buffer (990 µL, 100 mM, pH 7.5) was added and the mixture was sonicated in an ultrasonic bath (5 s) to ensure a fine distribution of the CN-OA-m. Then the substrate (10 mM, 10 µmol) dissolved in MeOH (1 M stock solution, 10.0 µL were used) was added, the crimp vial was sealed and the glass vial was irradiated in the photoreactor (455 nm, 1998 µmol photons m^{-2} s^{-1}) for 24 h at 30 °C and 500 rpm. Afterwards, the reaction was transferred to an Eppendorf tube (1.5 mL). Then isopropanol (15 µL, 194 µmol), NAD^+ (0.25 mg, 0.38 µmol) and ADH A (10 µL, 0.018 µg, stock: 1.75 µg/mL, 46 U/mg) were added and the reaction was incubated for 24 h at 30 °C and 600 rpm. Subsequently, the reaction was extracted with EtOAc (1x400 µL and 1x400µL + 10 mM 1-octanol) and the combined organic phases were dried over Na_2SO_4. The extracts were analysed by GC.

Photocatalytic Oxidation of Ethylbenzene with CN-OA-m in the Photoreactor for Photo-Chemo-Enzymatic Reactions

A crimp glass vial (1.5 mL) was charged with CN-OA-m (3.0 mg, 3.0 mg/mL) and five glass beads (1.0 mm, Merck). Then phosphate buffer (990 µL, 100 mM, pH 7.5) was added and the mixture was sonicated in an ultrasonic bath (5 s) to ensure a fine distribution of the CN-OA-m. Then the substrate (10 mM, 10 µmol) dissolved in MeOH (1 M stock solution, 10.0 µL were used) was added, the crimp vial was sealed and the glass vial was irradiated in the photoreactor (455 nm, 1998 µmol photons m^{-2} s^{-1}) for 24 h at 30 °C and 500 rpm. Subsequently, the reaction was extracted with EtOAc (1x400 µL and 1x400µL + 10 mM 1-octanol) and the combined organic phases were dried over Na_2SO_4. The extracts were analysed by GC.

ADH-A Catalysed Reduction of Acetophenone in the Presence of Hydrogen Peroxide

ADH-A (15 µL, 17.5 µg/mL) was added to phosphate buffer (100 mM, pH 7.5) containing hydrogen peroxide (0, 2 or 10 mM). The solution was incubated in a thermo shaker (30 min, 30 °C, 600 rpm). Afterwards, isopropanol (15 µL, 194 µmol), NAD^+ (0.25 mg, 0.38 µmol) and acetophenone (1.17 µL, 10 mM) were added and the reaction was incubated for 16 h at 30 °C and 600 rpm. Subsequently, the reaction was extracted with EtOAc (1x400 µL and 1x400µL + 10 mM 1-octanol) and the combined organic phases were dried over Na_2SO_4. The extracts were analysed by GC.

GC Analytics

Table S5. Information about the GC measurements.

Substrate	Column[a]	Retention time T_R/[min]	Temperature program
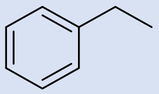<br>ethylbenzene	Quantification:<br>Column A<br><br>Enantiomeric excess:<br>Column B	Quantification:<br>ethylbenzene 1.79<br>acetophenone 5.95<br>1-phenylethanol 6.72<br><br>Enantiomeric excess:<br>(*R*)-1-phenylethanol 8.96<br>(*S*)-1-phenylethanol 9.40	Quantification:<br>70 °C hold 2 min, 20 °C/min to 140 °C hold 1 min, 20 °C/min to 250 °C hold 2.5 min.<br><br>Enantiomeric excess:<br>70 °C hold 0.5 min, 20 °C/min to 120 °C hold 8 min, 20 °C/min to 180 °C hold 1 min.
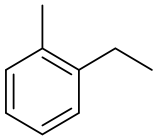<br>1-ethyl-2-methylbenzene	Quantification:<br>Column A<br><br>Enantiomeric excess:<br>Column B	Quantification:<br>1-ethyl-2-methylbenzene 2.66<br>1-(*o*-tolyl)ethan-1-one 6.13<br>1-(*o*-tolyl)ethan-1-ol 7.72<br>(2-ethylphenyl)methanol 8.58<br><br>Enantiomeric excess:<br>(*R*)-1-(*o*-tolyl)ethan-1-ol 13.01<br>(*S*)-1-(*o*-tolyl)ethan-1-ol 13.92	Quantification:<br>70 °C hold 2 min, 20 °C/min to 140 °C hold 1 min, 20 °C/min to 200 °C hold 4 min, 10 °C/min to 210 °C hold 4 min, 20 °C/min to 250 °C hold 2 min.<br><br>Enantiomeric excess:<br>70 °C hold 0.5 min, 20 °C/min to 120 °C hold 8 min, 20 °C/min to 140 °C hold 3 min, 20 °C/min to 160 °C hold 3 min, 20 °C/min to 180 °C hold 3 min.
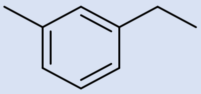<br>1-ethyl-3-methylbenzene	Quantification:<br>Column A<br><br>Enantiomeric excess:<br>Column B	Quantification:<br>1-ethyl-2-methylbenzene 2.37<br>1-(*m*-tolyl)ethan-1-one 6.74<br>1-(*m*-tolyl)ethan-1-ol 7.45<br>(3-ethylphenyl)methanol 8.54<br><br>Enantiomeric excess:<br>(*R*)-1-(*m*-tolyl)ethan-1-ol 12.00<br>(*S*)-1-(*m*-tolyl)ethan-1-ol 12.28	Quantification:<br>70 °C hold 0.5 min, 20 °C/min to 120 °C hold 1 min, 20 °C/min to 200 °C hold 4 min, 10 °C/min to 210 °C hold 4 min, 20 °C/min to 250 °C hold 2 min.<br><br>Enantiomeric excess:<br>70 °C hold 0.5 min, 20 °C/min to 120 °C hold 8 min, 20 °C/min to 140 °C hold 3 min, 20 °C/min to 160 °C hold 3 min, 20 °C/min to 180 °C hold 3 min.
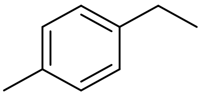<br>1-ethyl-4-methylbenzene	Quantification:<br>Column A<br><br>Enantiomeric excess:<br>Column B	Quantification:<br>1-ethyl-2-methylbenzene 2.36<br>1-(*p*-tolyl)ethan-1-one 6.70<br>1-(*p*-tolyl)ethan-1-ol 7.46<br>(4-ethylphenyl)methanol 8.56<br><br>Enantiomeric excess:<br>(*R*)-1-(*p*-tolyl)ethan-1-ol 11.46<br>(*S*)-1-(*p*-tolyl)ethan-1-ol 11.99	Quantification:<br>70 °C hold 2 min, 20 °C/min to 140 °C hold 1 min, 20 °C/min to 200 °C hold 4 min, 10 °C/min to 210 °C hold 4 min, 20 °C/min to 250 °C hold 2 min.<br><br>Enantiomeric excess:<br>70 °C hold 0.5 min, 20 °C/min to 120 °C hold 8 min, 20 °C/min to 140 °C hold 3 min, 20 °C/min to 160 °C hold 3 min, 20 °C/min to 180 °C hold 3 min.
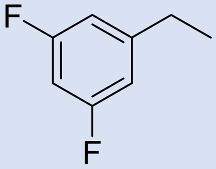<br>1-ethyl-3,5-difluorobenzene	Quantification:<br>Column A<br><br>Enantiomeric excess:<br>Column B	Quantification:<br>1-ethyl-3,5-difluorobenzene 1.85<br>1-(3,5-difluorophenyl)ethan-1-one 5.15<br>1-(3,5-difluorophenyl)ethan-1-ol 7.08<br><br>Enantiomeric excess:<br>(*R*)-1-(3,5-difluorophenyl)ethan-1-ol 13.70<br>(*S*)-1-(3,5-difluorophenyl)ethan-1-ol 14.01	Quantification:<br>70 °C hold 2 min, 20 °C/min to 140 °C hold 1 min, 20 °C/min to 200 °C hold 4 min, 10 °C/min to 210 °C hold 4 min, 20 °C/min to 250 °C hold 2 min.<br><br>Enantiomeric excess:<br>70 °C hold 0.5 min, 20 °C/min to 110 °C hold 6 min, 10 °C/min to 115 °C hold 4 min, 10 °C/min to 120 °C hold 3 min, 20 °C/min to 180 °C hold 1 min.

**Substrate**	Column[a]	Retention time T_R/[min]	Temperature program
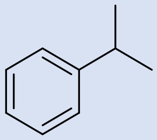<br>cumene	Quantification:<br>Column A	Quantification:<br>cumene 1.92<br>2-phenylpropan-2-ol 6.31	Quantification:<br>70 °C hold 2 min, 20 °C/min to 140 °C hold 1 min, 20 °C/min to 250 °C hold 2.5 min.
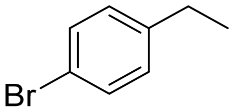<br>1-bromo-4-ethylbenzen	Quantification:<br>Column A<br><br>Enantiomeric excess:<br>Column B	Quantification:<br>1-bromo-4-ethylbenzen 4.88<br>1-(4-bromophenyl)ethan-1-one 8.94<br>1-(4-bromophenyl)ethan-1-ol 9.95<br><br>Enantiomeric excess:<br>(*R*)-1-(4-bromophenyl)ethan-1-ol 13.03<br>(*S*)-1-(4-bromophenyl)ethan-1-ol 13.41	Quantification:<br>70 °C hold 2 min, 20 °C/min to 140 °C hold 1 min, 20 °C/min to 200 °C hold 4 min, 10 °C/min to 210 °C hold 4 min, 20 °C/min to 250 °C hold 2 min.<br><br>Enantiomeric excess:<br>70 °C hold 0.5 min, 20 °C/min to 140 °C hold 6 min, 20 °C/min to 160 °C hold 6 min, 20 °C/min to 180 °C hold 1 min.
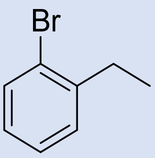<br>1-bromo-2-ethylbenzen	Quantification:<br>Column A<br><br>Enantiomeric excess:<br>Column B	Quantification:<br>1-bromo-2-ethylbenzen 4.62<br>1-(2-bromophenyl)ethan-1-one 8.46<br>1-(2-bromophenyl)ethan-1-ol 9.44<br><br>Enantiomeric excess:<br>(*R*)-1-(2-bromophenyl)ethan-1-ol 12.45<br>(*S*)-1-(2-bromophenyl)ethan-1-ol 13.46	Quantification:<br>70 °C hold 2 min, 20 °C/min to 140 °C hold 1 min, 20 °C/min to 200 °C hold 4 min, 10 °C/min to 210 °C hold 4 min, 20 °C/min to 250 °C hold 2 min.<br><br>Enantiomeric excess:<br>70 °C hold 0.5 min, 20 °C/min to 140 °C hold 6 min, 20 °C/min to 160 °C hold 6 min, 20 °C/min to 180 °C hold 1 min.
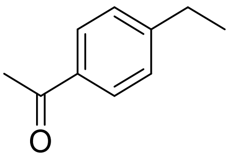<br>1-(4-ethylphenyl)ethan-1-one	Quantification:<br>Column A<br><br>Enantiomeric excess:<br>Column B	Quantification:<br>1-(4-ethylphenyl)ethan-1-one 7.63<br>1,1'-(1,4-phenylene)bis(ethan-1-one) 11.14<br>1-(4-(1-hydroxyethyl)phenyl)ethan-1-one 12.84<br><br>Enantiomeric excess:<br>(*R*)-1-(4-(1-hydroxyethyl)phenyl)ethan-1-one 21.34<br>(*S*)-1-(4-(1-hydroxyethyl)phenyl)ethan-1-one 21.55	Quantification:<br>70 °C hold 2 min, 20 °C/min to 140 °C hold 1 min, 20 °C/min to 200 °C hold 4 min, 10 °C/min to 210 °C hold 4 min, 20 °C/min to 250 °C hold 2 min.<br><br>Enantiomeric excess:<br>70 °C hold 0.5 min, 20 °C/min to 120 °C hold 8 min, 20 °C/min to 140 °C hold 3 min, 20 °C/min to 160 °C hold 3 min, 20 °C/min to 180 °C hold 3 min.
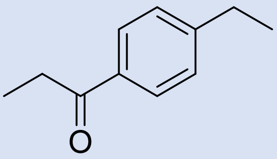<br>1-(4-ethylphenyl)propan-1-one	Quantification:<br>Column A<br><br>Enantiomeric excess:<br>Column B	Quantification:<br>1-(4-ethylphenyl)propan-1-one 8.07<br>1-(4-acetylphenyl)propan-1-one 11.67<br>1-(4-(1-hydroxyethyl)phenyl)propan-1-one 13.55<br><br>Enantiomeric excess:<br>(*R*)-1-(4-(1-hydroxyethyl)phenyl)propan-1-one 22.98<br>(*S*)-1-(4-(1-hydroxyethyl)phenyl)propan-1-one 23.2l*	Quantification:<br>70 °C hold 2 min, 20 °C/min to 140 °C hold 1 min, 20 °C/min to 200 °C hold 4 min, 10 °C/min to 210 °C hold 4 min, 20 °C/min to 250 °C hold 2 min.<br><br>Enantiomeric excess:<br>70 °C hold 0.5 min, 20 °C/min to 120 °C hold 8 min, 20 °C/min to 140 °C hold 3 min, 20 °C/min to 160 °C hold 3 min, 20 °C/min to 180 °C hold 6 min, 20 °C/min to 200 °C hold 3 min.

[a] Column A CP WAX 52CB; column B CP-ChiraSil-DEX CB; *no reference material for the (*S*)-enantiomer; 5 mM 1-octanol were used as internal standard.

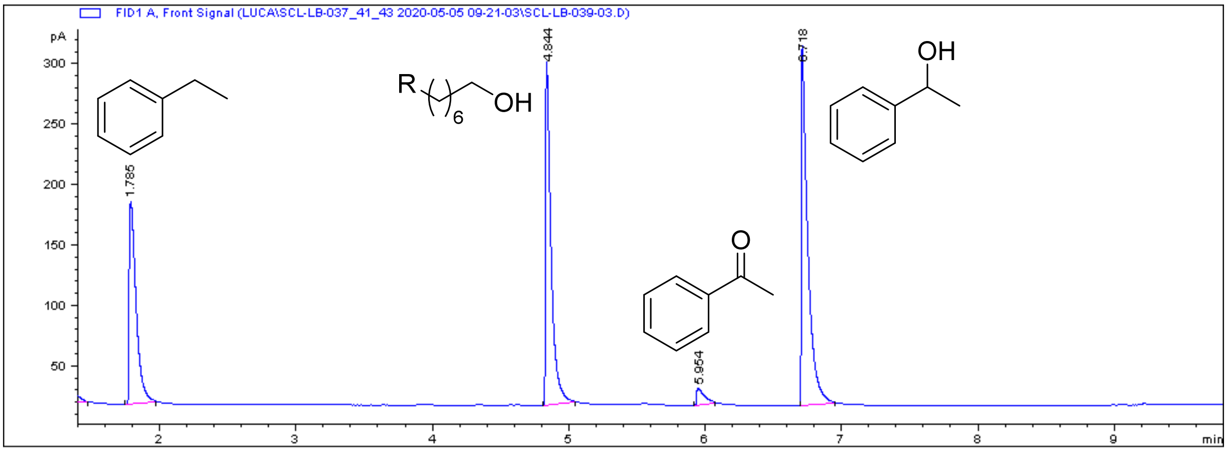

Figure S6. GC chromatogram of the photochemoenzymatic hydroxylation of ethylbenzene to 1-phenylethanol.

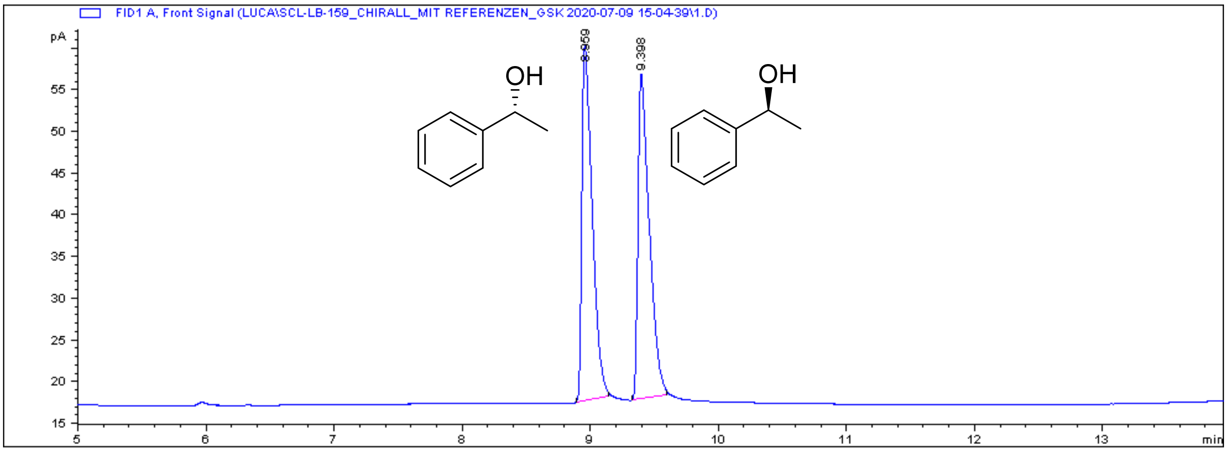

Figure S7. Racemic reference material of 1-phenylethanol.

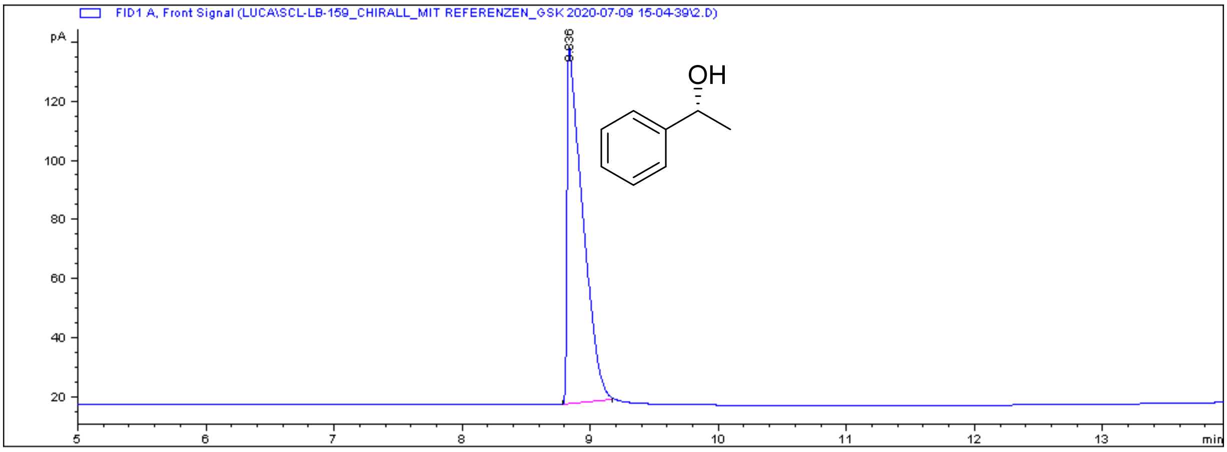

Figure S8. Reference material of the (*R*)-enantiomer of 1-phenylethanol.

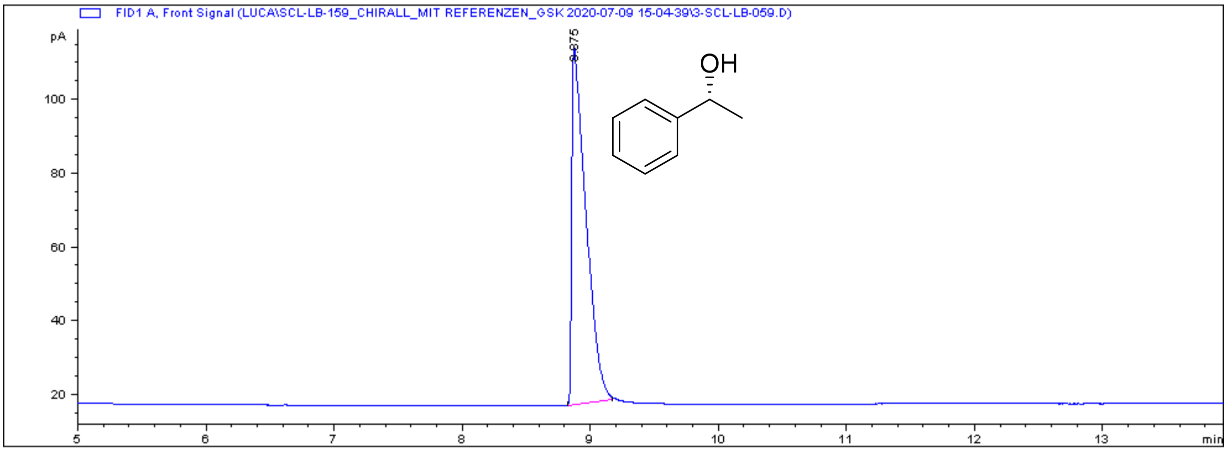

Figure S9. GC chromatogram of the isolated and purified (*R*)-1-phenylethanol.

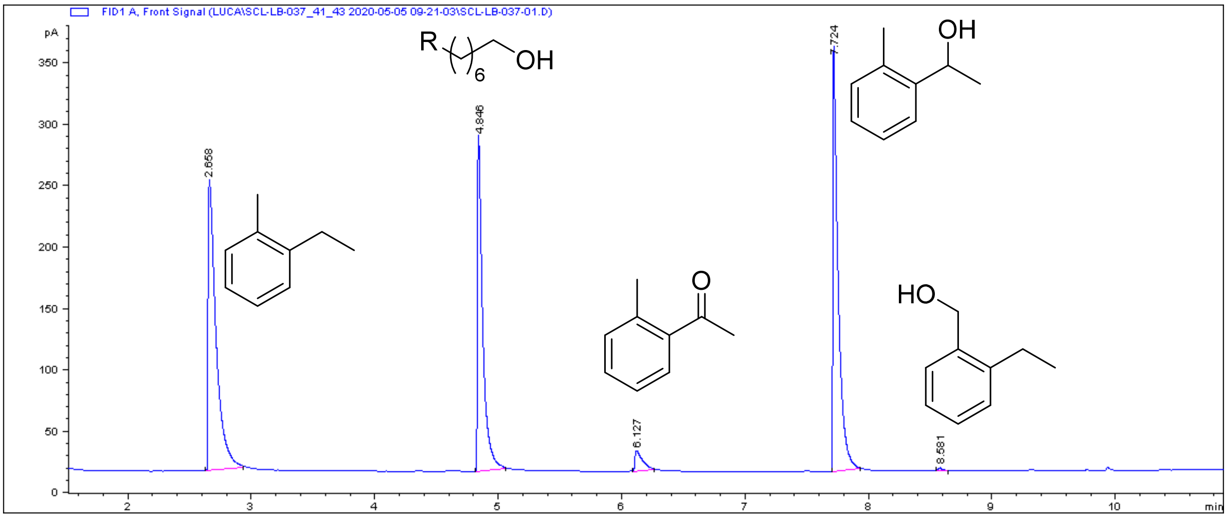

Figure S10. GC chromatogram of the photochemoenzymatic hydroxylation of 1-ethyl-2-methylbenzene to 1-(*o*-tolyl)ethan-1-ol.

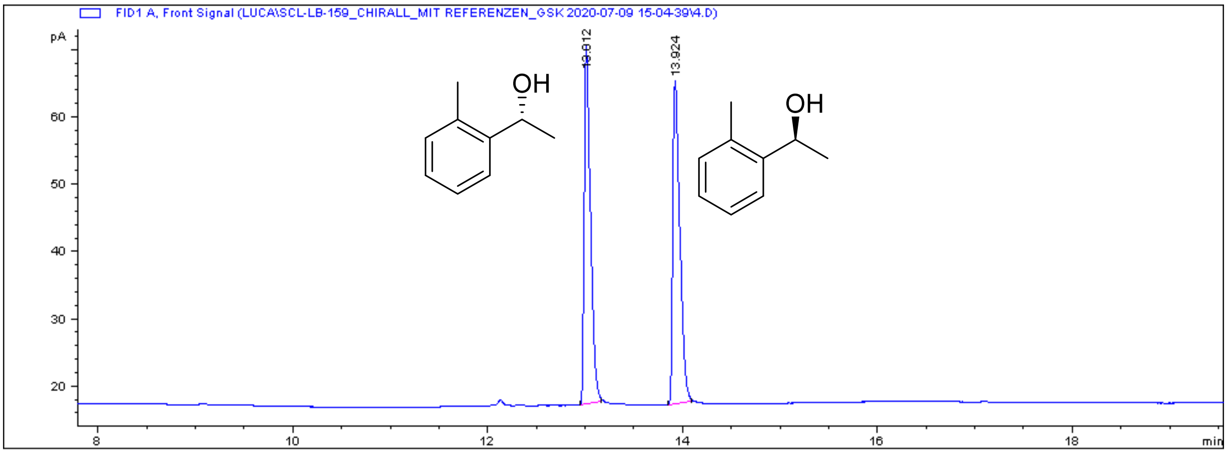

Figure S11. Racemic reference material of 1-(*o*-tolyl)ethan-1-ol.

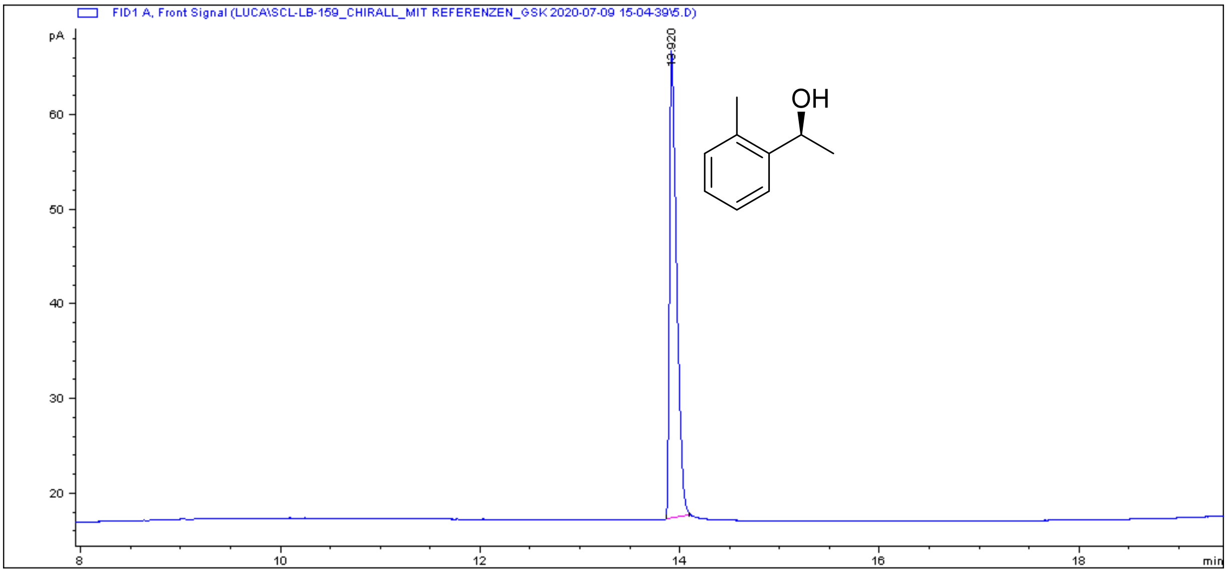

Figure S12. Reference material of the (*S*)-enantiomer of 1-(*o*-tolyl)ethan-1-ol.

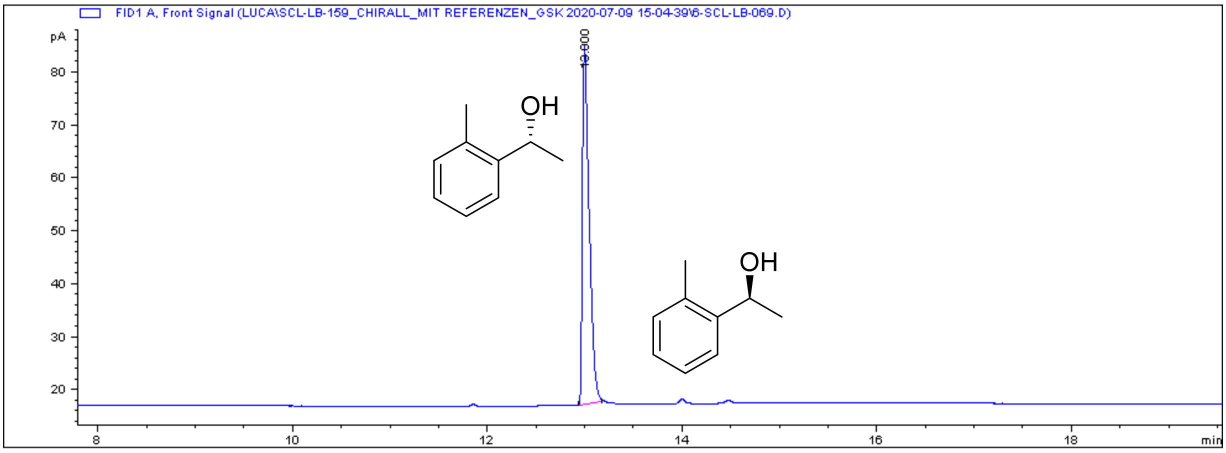

Figure S13. GC chromatogram of the isolated and purified (*R*)-1-(*o*-tolyl)ethan-1-ol.

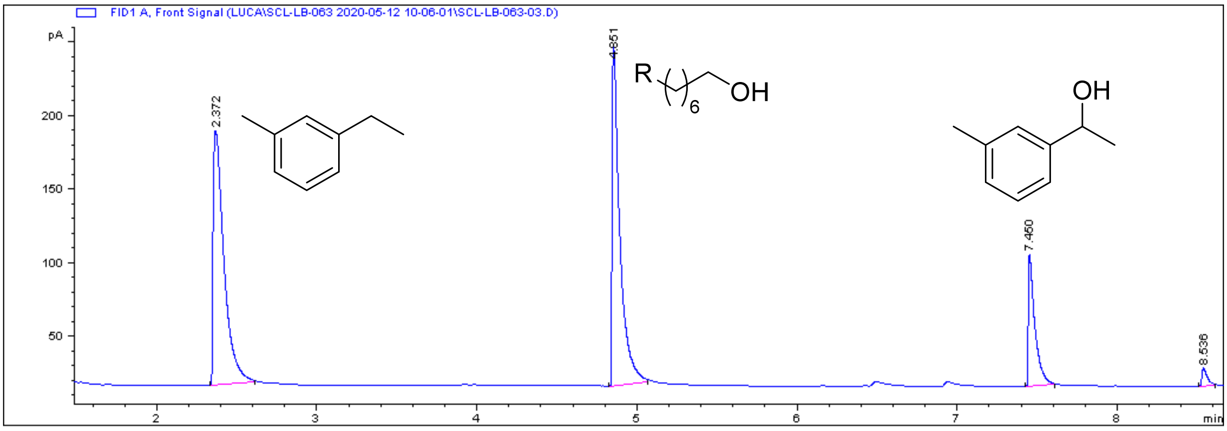

Figure S14. GC chromatogram of the photo-chemo-enzymatic hydroxylation of 1-ethyl-3-methylbenzene to 1-(*m*-tolyl)ethan-1-ol.

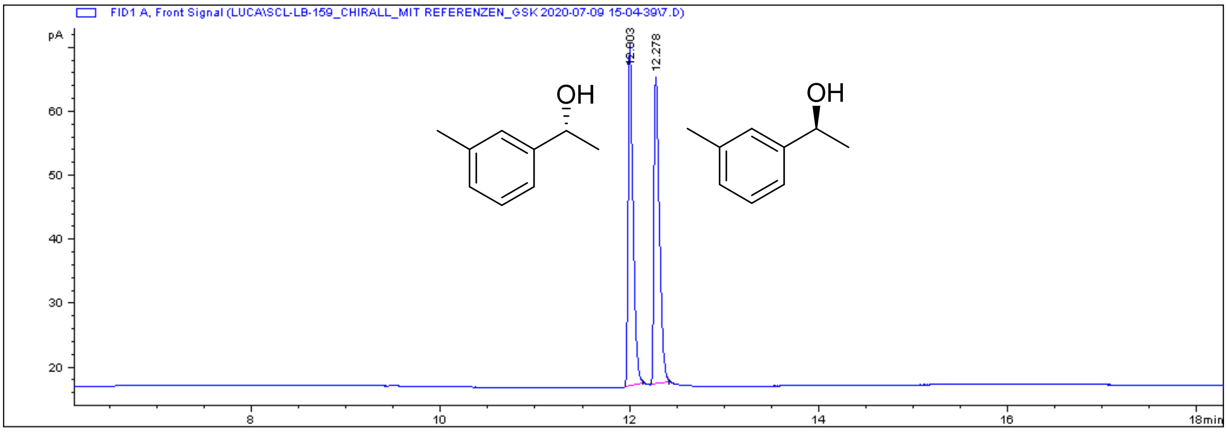

Figure S15. Racemic reference material of 1-(*m*-tolyl)ethan-1-ol.

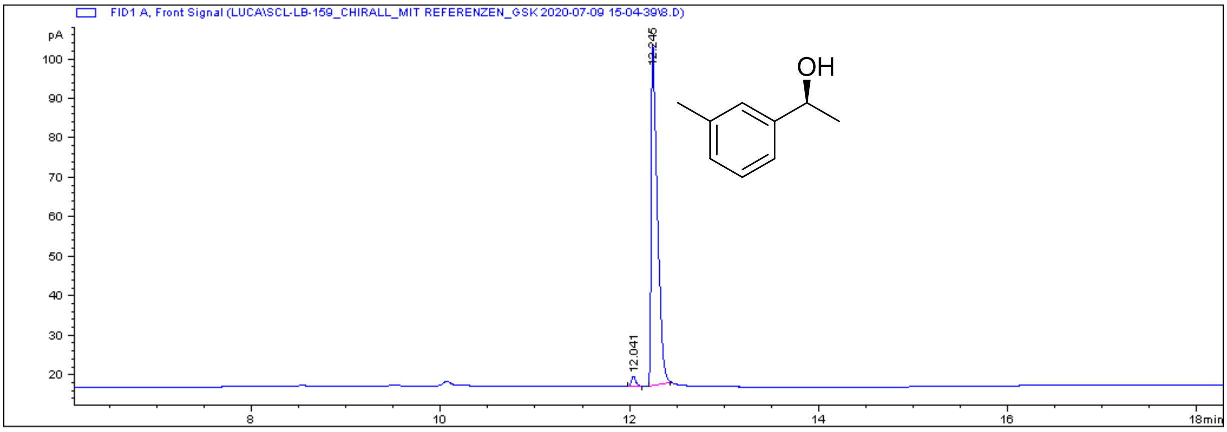

Figure S16. Reference material of the (*S*)-enantiomer of 1-(*m*-tolyl)ethan-1-ol.

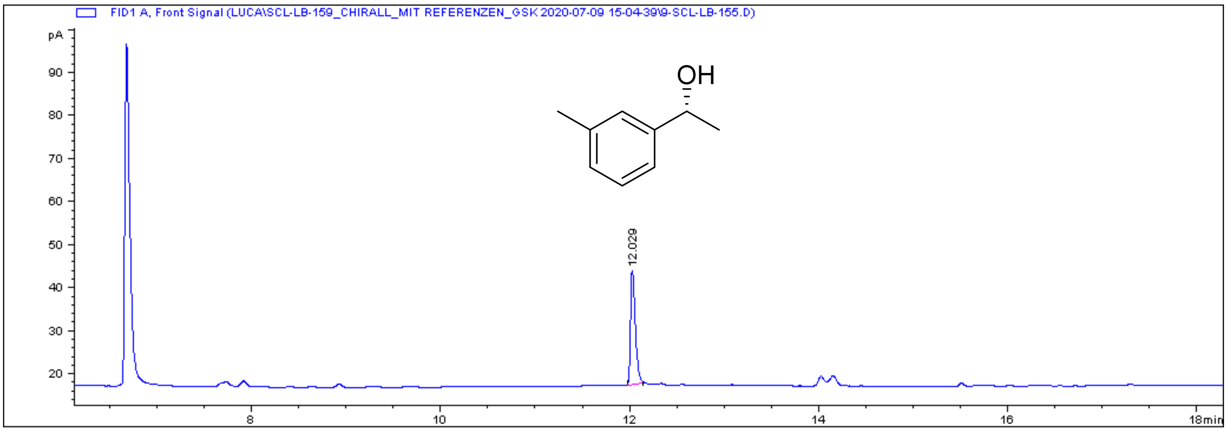

Figure S17. GC chromatogram of the isolated and purified (*R*)-1-(*m*-tolyl)ethan-1-ol.

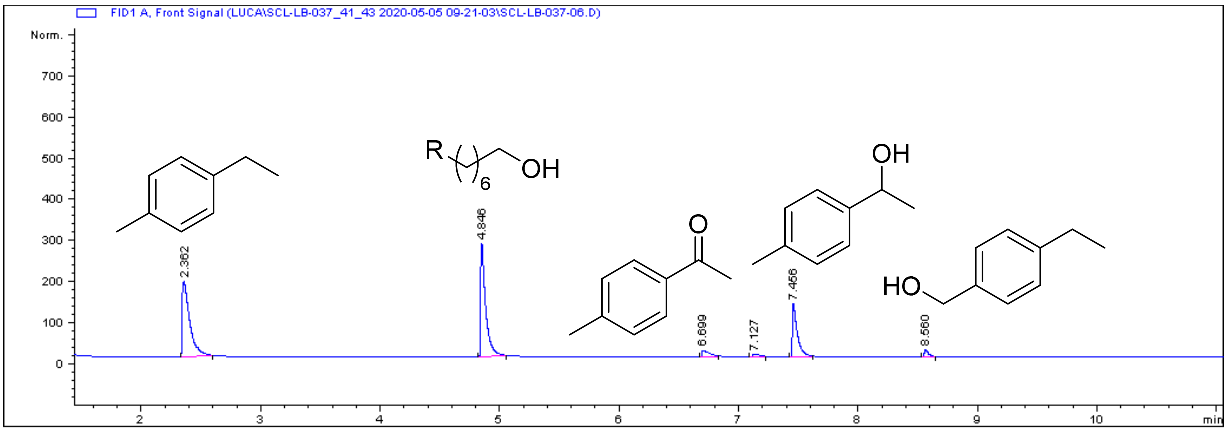

Figure S18. GC chromatogram of the photo-chemo-enzymatic hydroxylation of 1-ethyl-4-methylbenzene to 1-(*p*-tolyl)ethan-1-ol.

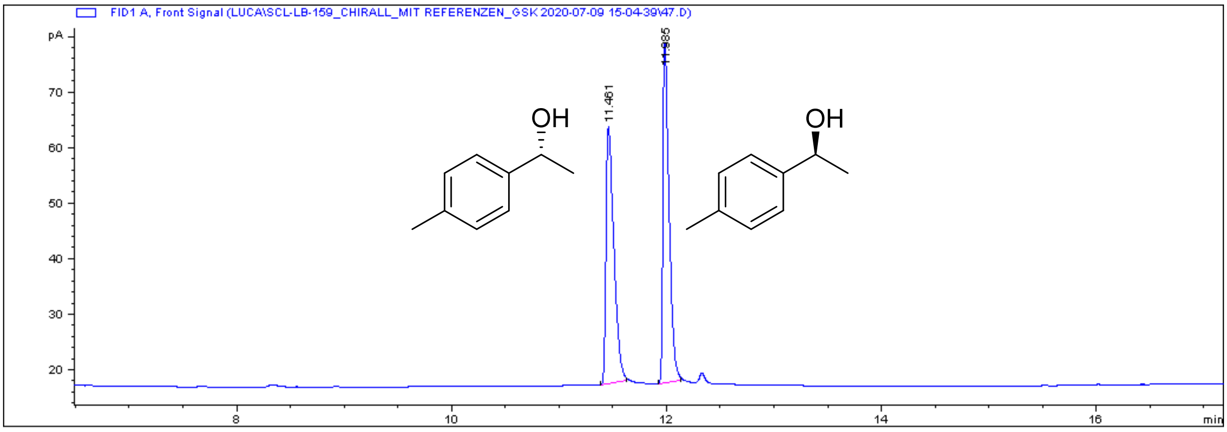

Figure S19. Racemic reference material of 1-(*p*-tolyl)ethan-1-ol.

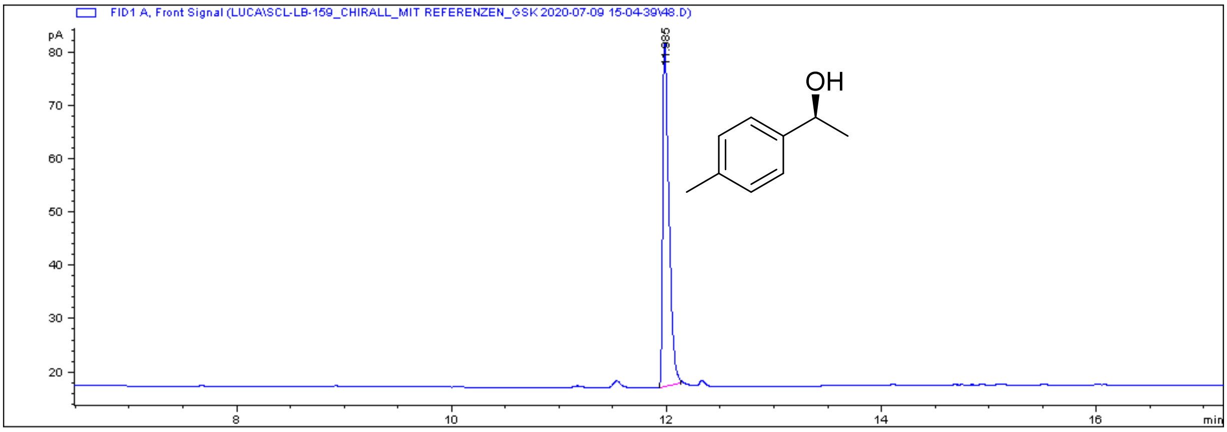

Figure S20. Reference material of the (*S*)-enantiomer of 1-(*p*-tolyl)ethan-1-ol.

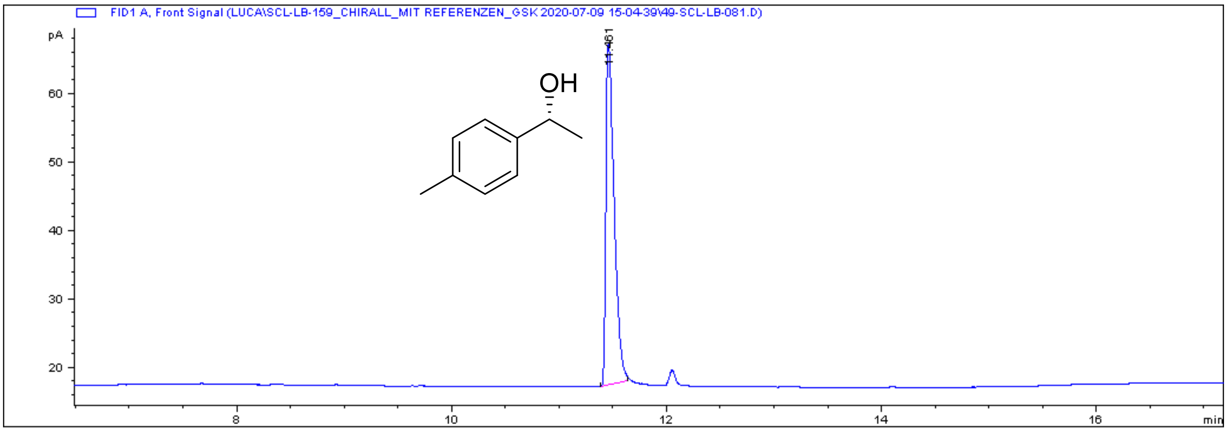

Figure S21. GC chromatogram of the isolated and purified (*R*)-1-(*p*-tolyl)ethan-1-ol.

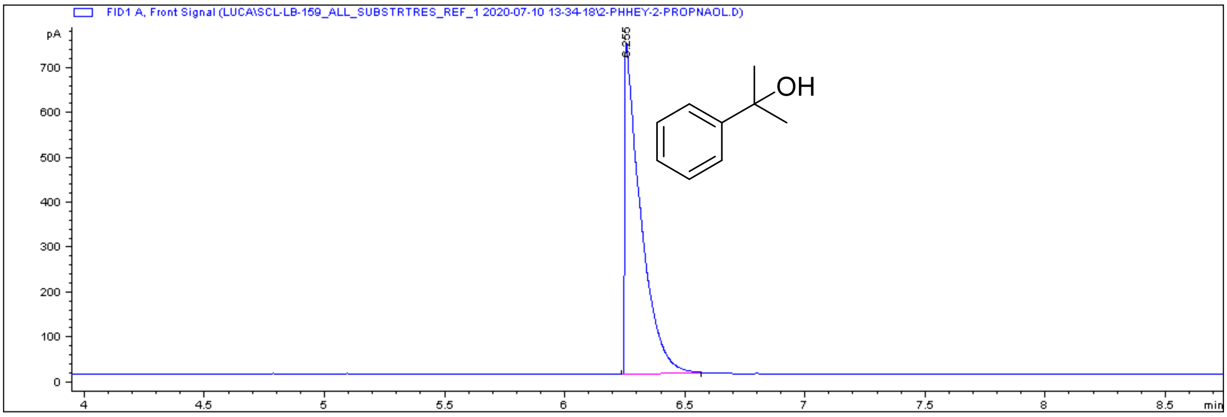

Figure S22. Reference material of 2-phenylpropan-2-ol.

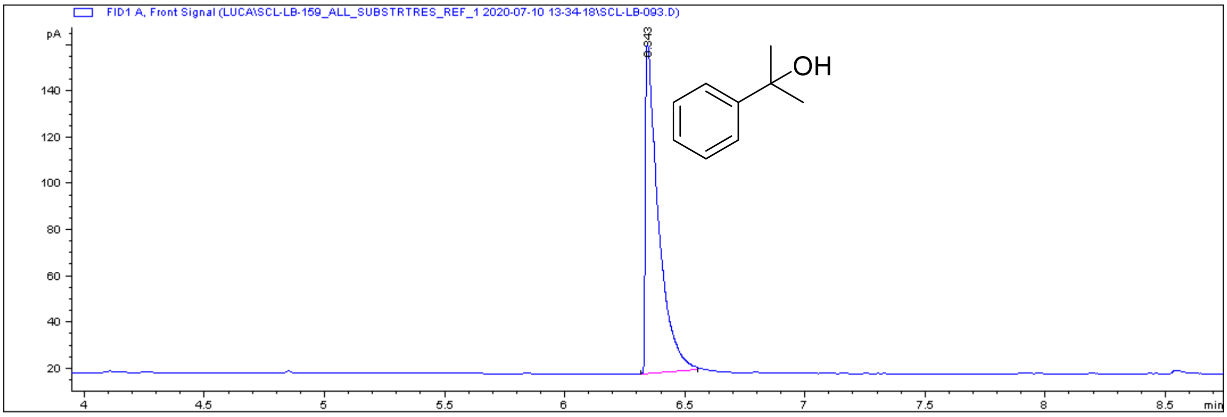

Figure S23. GC chromatogram of the isolated and purified 2-phenylpropan-2-ol.

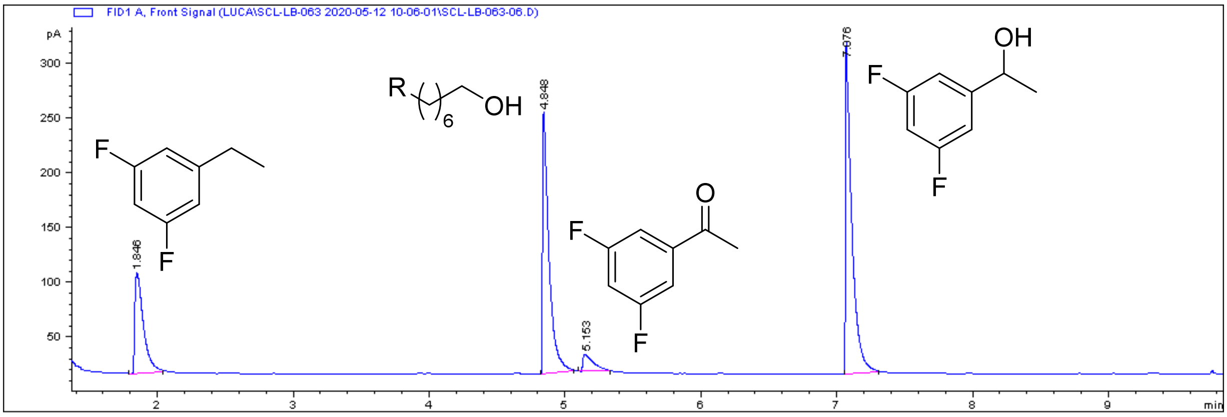

Figure S24. GC chromatogram of the photo-chemo-enzymatic hydroxylation of 1-ethyl-3,5-difluorobenzene to 1-(3,5-difluorophenyl)ethan-1-ol.

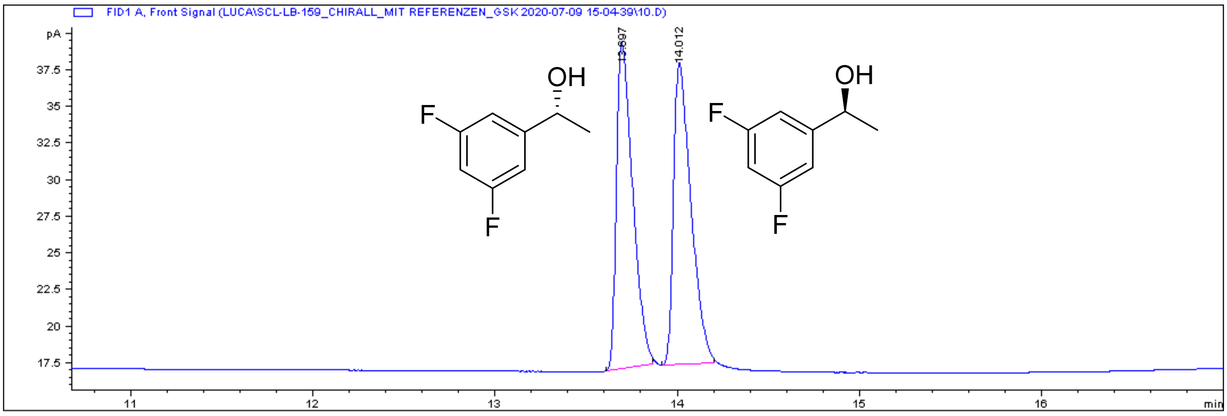

Figure S25. Racemic reference material of 1-(3,5-difluorophenyl)ethan-1-ol.

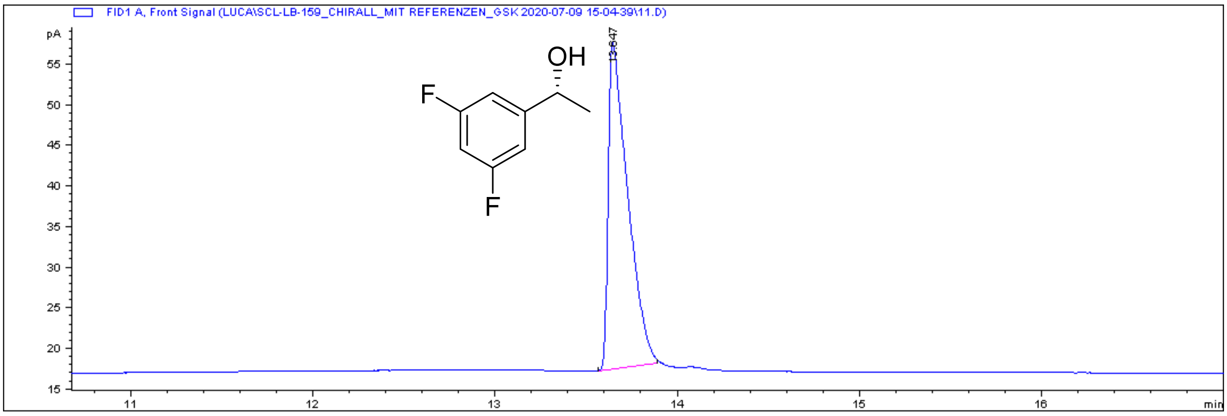

Figure S26. Reference material of the (*R*)-enantiomer of 1-(3,5-difluorophenyl)ethan-1-ol.

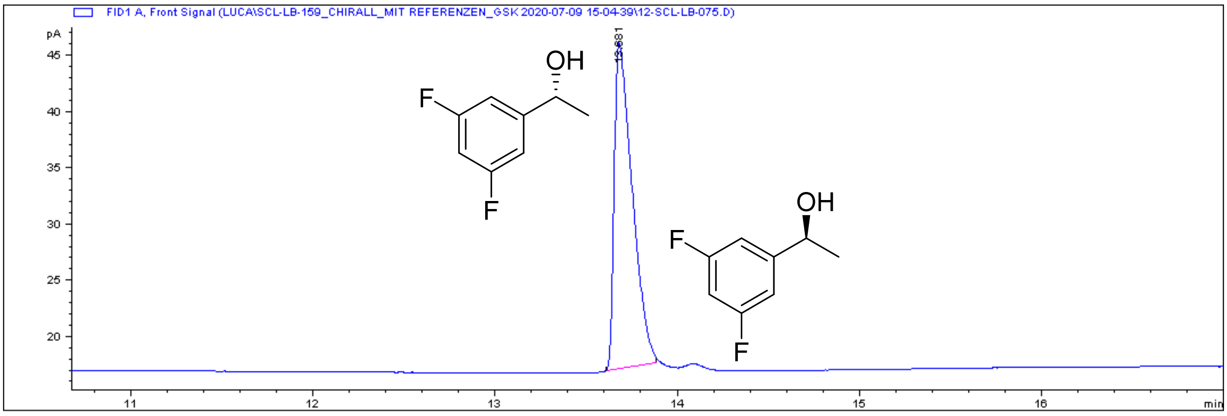

Figure S27. GC chromatogram of the isolated and purified (*R*)-1-(3,5-difluorophenyl)ethan-1-ol.

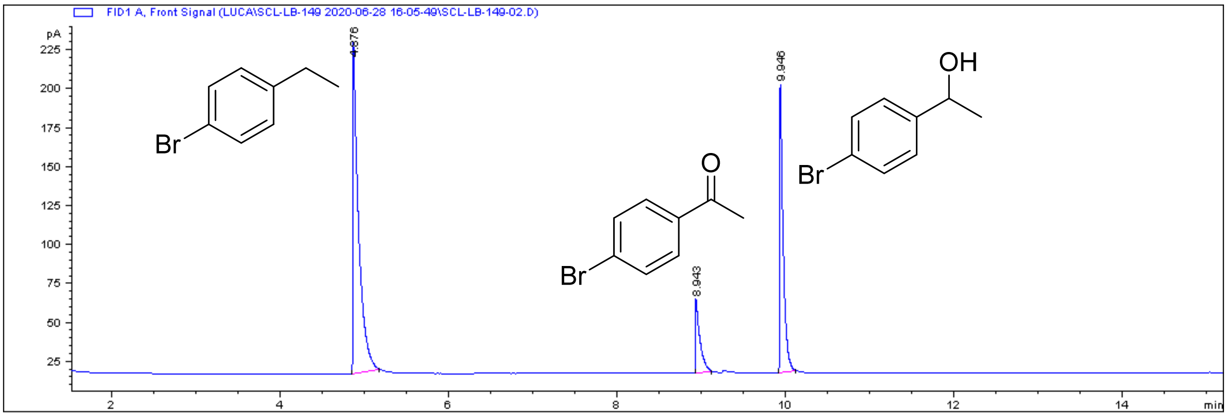

Figure S28. GC chromatogram of the photo-chemo-enzymatic hydroxylation of 1-bromo-4-ethylbenzen to 1-(4-bromophenyl)ethan-1-ol.

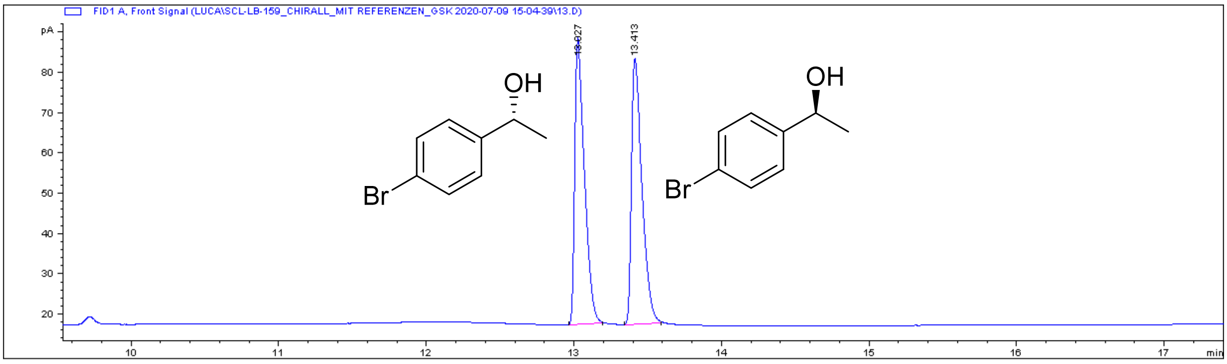

Figure S29. Racemic reference material of 1-(4-bromophenyl)ethan-1-ol.

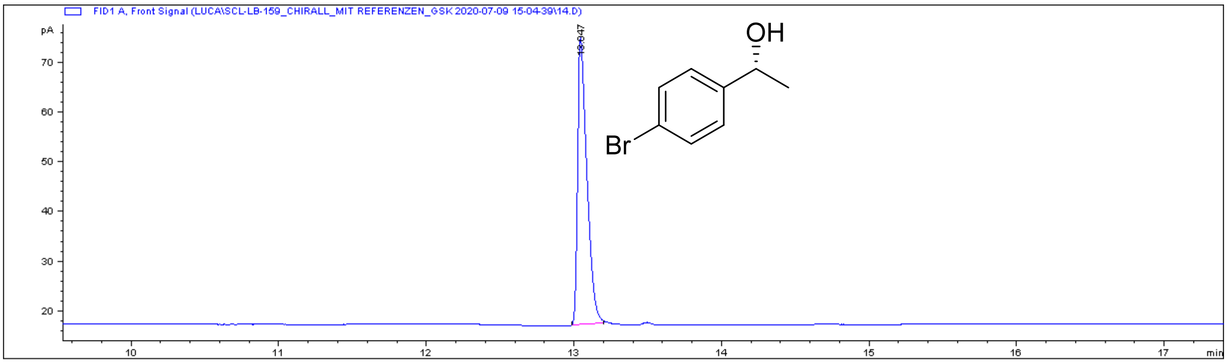

Figure S30. Reference material of the (*R*)-enantiomer of 1-(4-bromophenyl)ethan-1-ol.

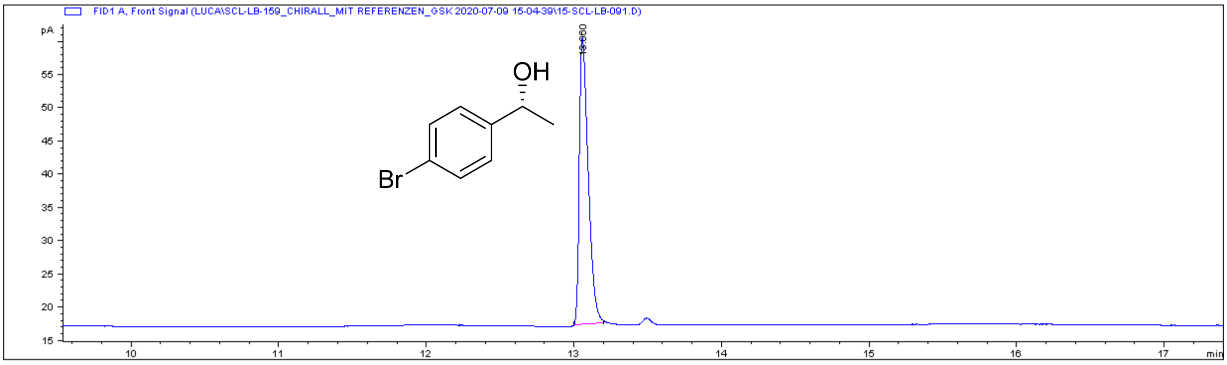

Figure S31. GC chromatogram of the isolated and purified (*R*)-1-(4-bromophenyl)ethan-1-ol.

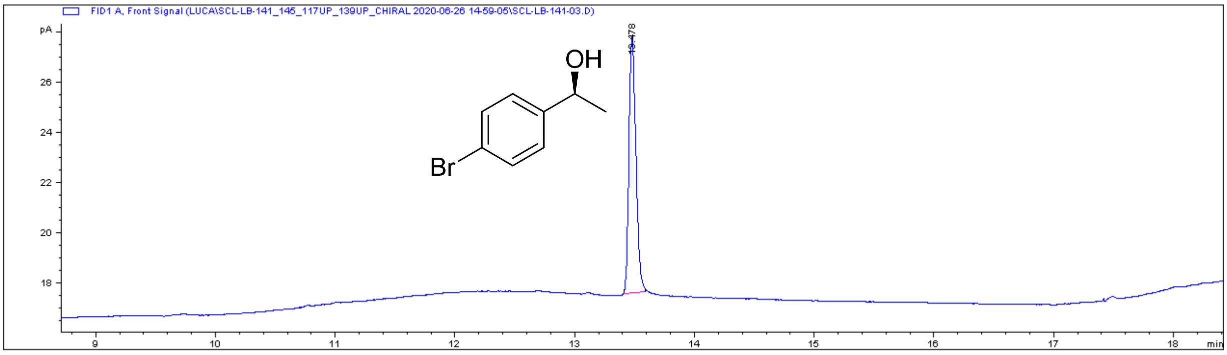

Figure S32. GC chromatogram of the (*S*)-1-(4-bromophenyl)ethan-1-ol obtained through the CN-OA-m/ADH-A cascade.

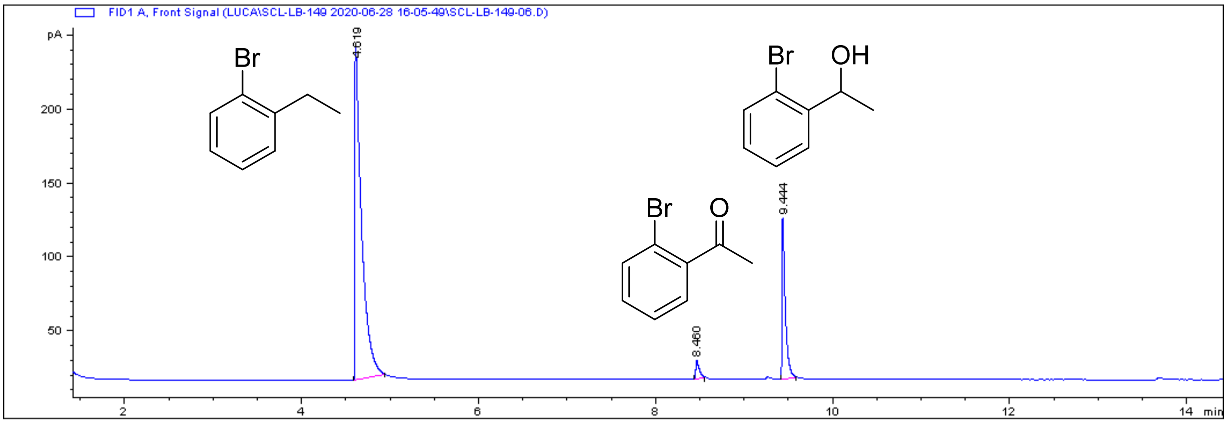

Figure S33. GC chromatogram of the photo-chemo-enzymatic hydroxylation of 1-bromo-2-ethylbenzen to 1-(2-bromophenyl)ethan-1-ol.

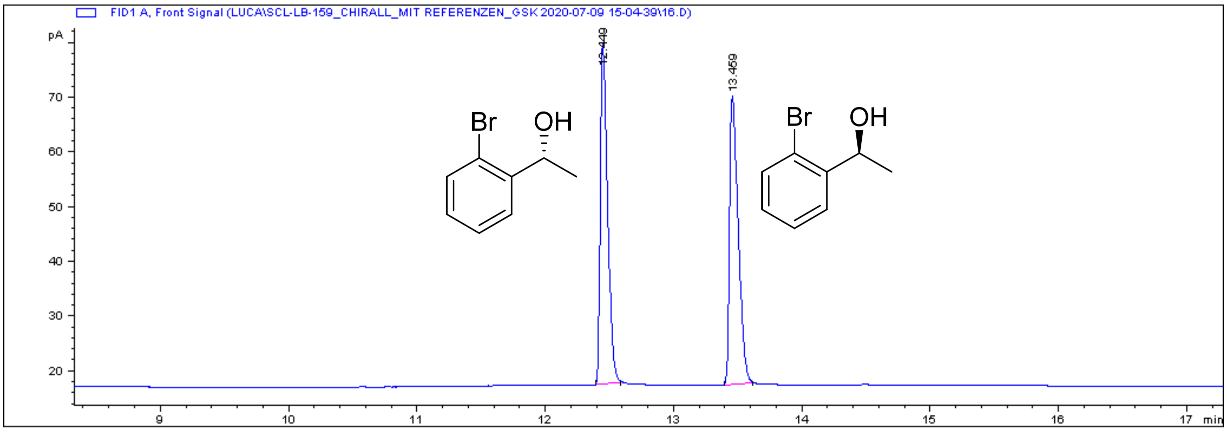

Figure S34. Racemic reference material of 1-(2-bromophenyl)ethan-1-ol.

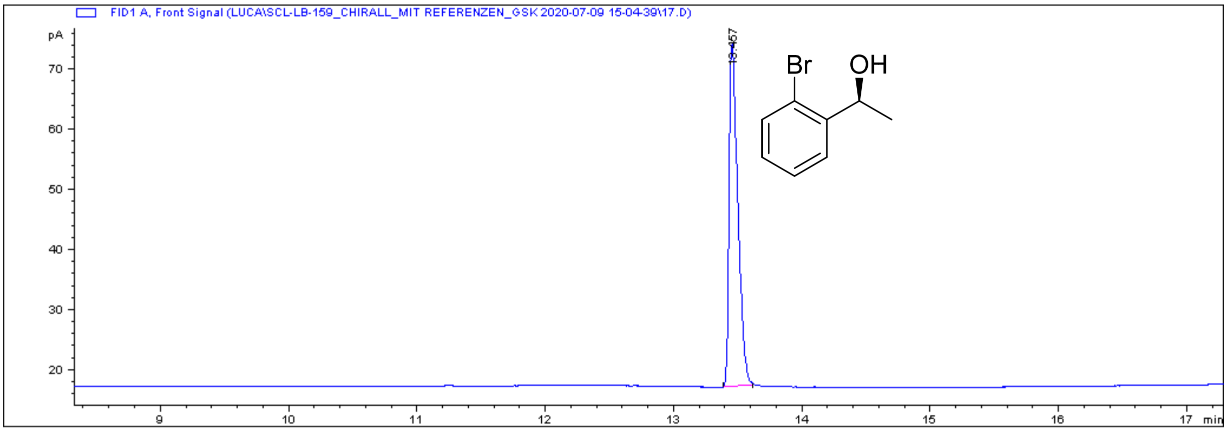

Figure S35. Reference material of the (*S*)-enantiomer of 1-(2-bromophenyl)ethan-1-ol.

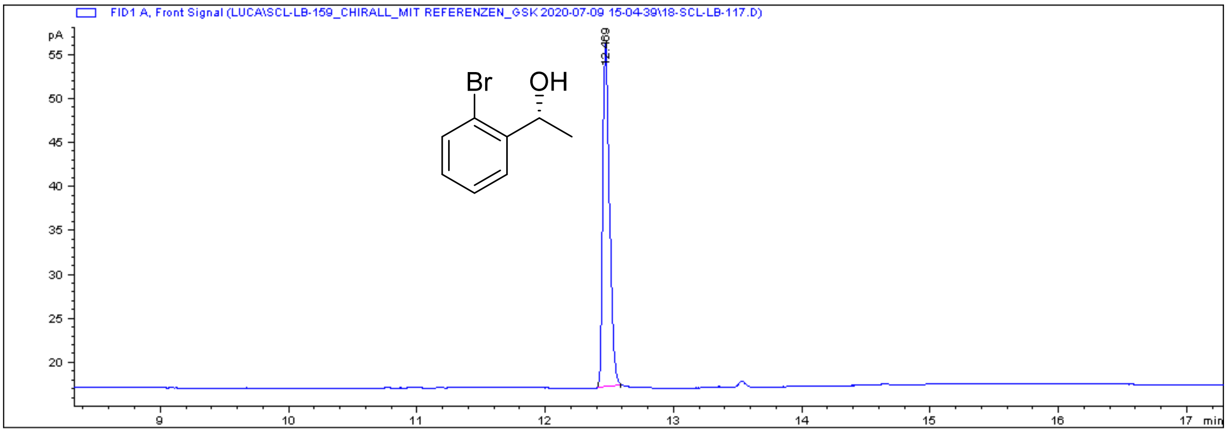

Figure S36. GC chromatogram of the isolated and purified (*R*)-1-(2-bromophenyl)ethan-1-ol.

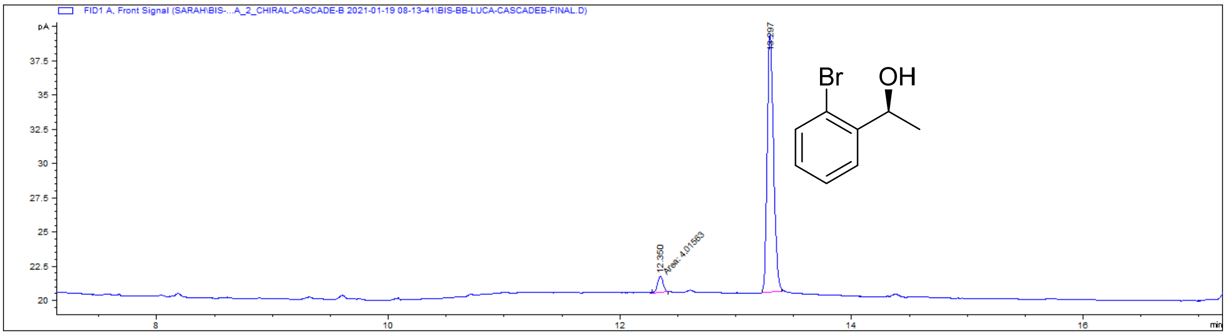

Figure S37. GC chromatogram of the (*S*)-1-(2-bromophenyl)ethan-1-ol obtained through the CN-OA-m/ADH-A cascade.

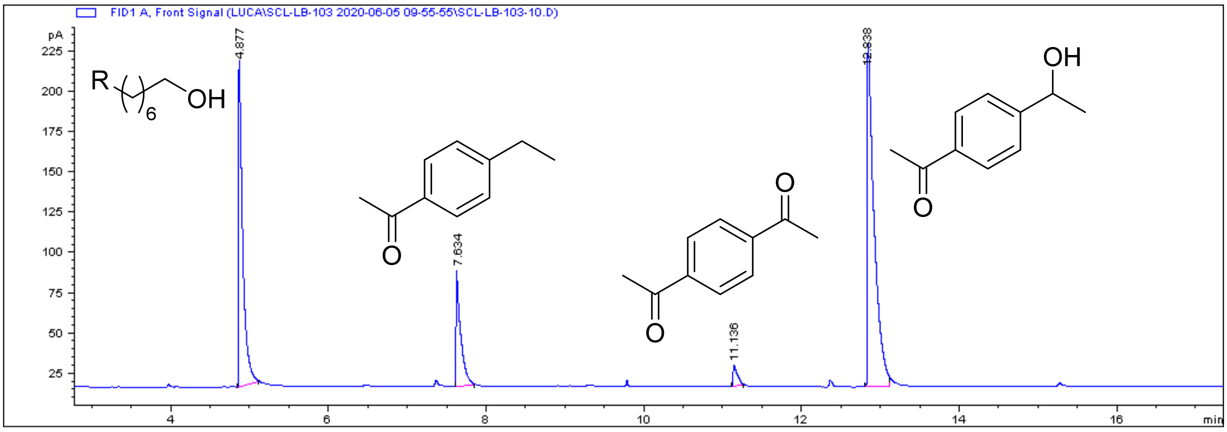

Figure S38. GC chromatogram of the photo-chemo-enzymatic hydroxylation of 1-(4-ethylphenyl)ethan-1-one to 1-(4-(1-hydroxyethyl)phenyl)ethan-1-one.

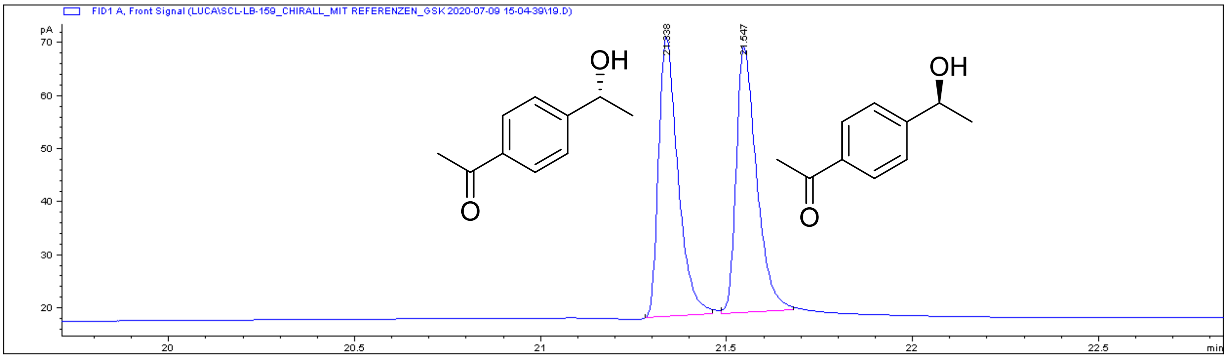

Figure S39. Racemic reference material of 1-(4-(1-hydroxyethyl)phenyl)ethan-1-one.

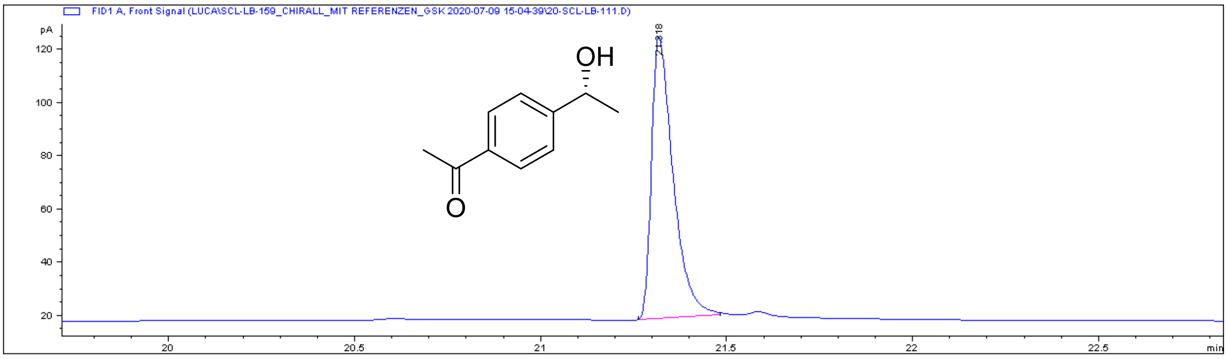

Figure S40. GC chromatogram of the isolated and purified 1-(4-(1-hydroxyethyl)phenyl)ethan-1-one.

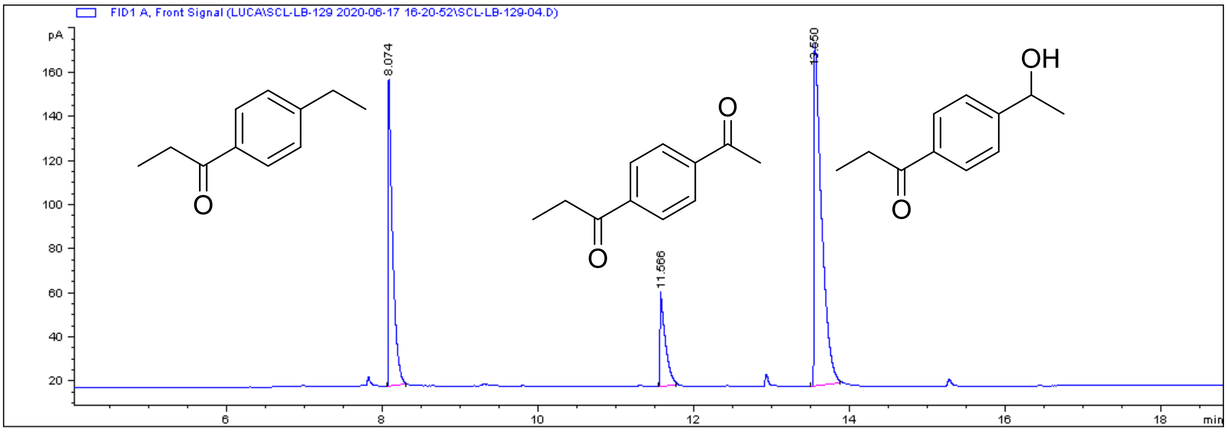

Figure S41. GC chromatogram of the photo-chemo-enzymatic hydroxylation of 1-(4-ethylphenyl)propan-1-one to 1-(4-(1-hydroxyethyl)phenyl)propan-1-one.

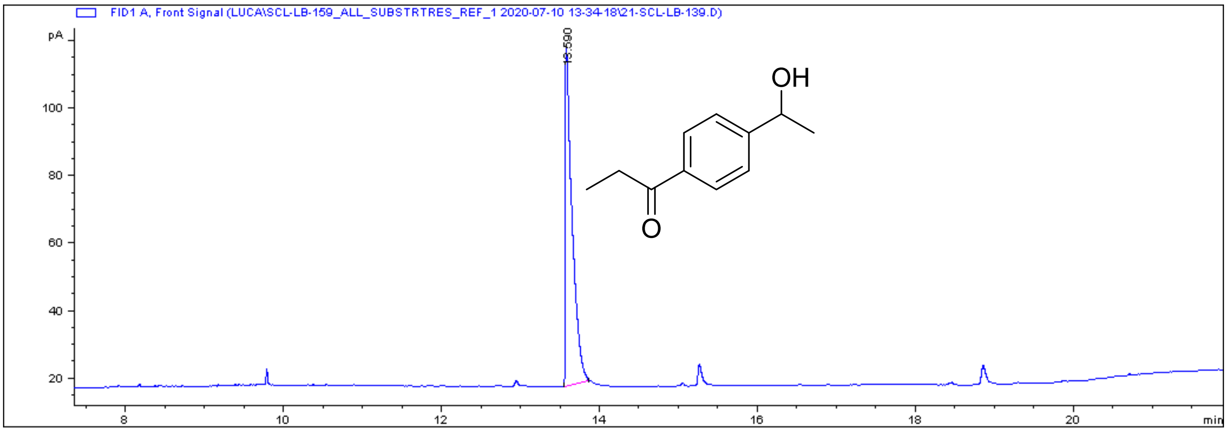

Figure S42. GC chromatogram of the isolated and purified 1-(4-(1-hydroxyethyl)phenyl)propan-1-one.

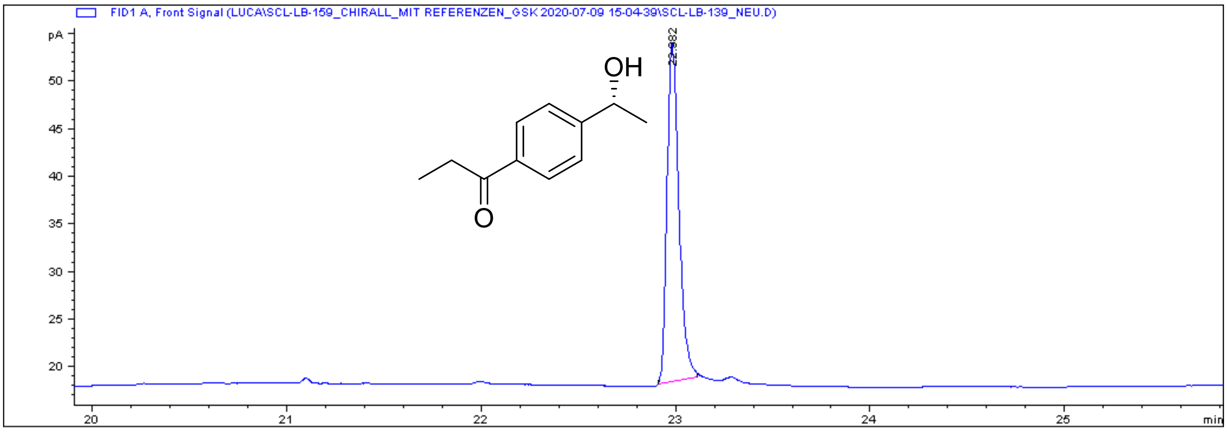

Figure S43. GC chromatogram of the isolated and purified (*R*)-1-(4-(1-hydroxyethyl)phenyl)propan-1-one.

Additional Experiments

Influence of the MeOH Concentration

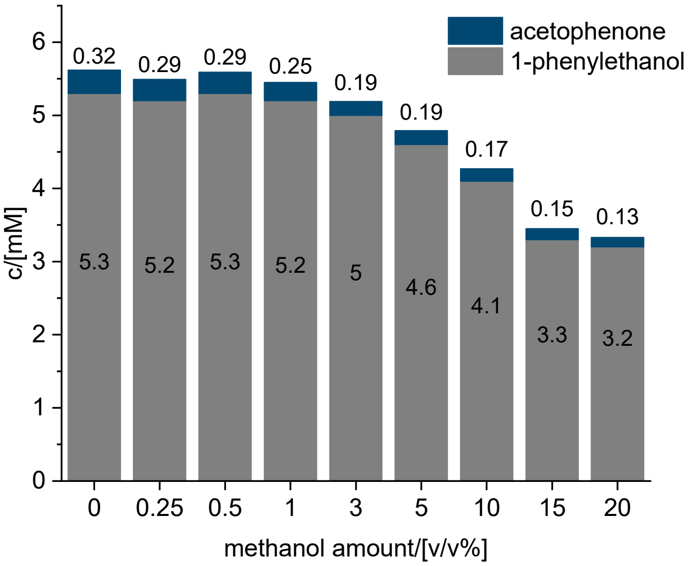

Figure S44. Influence of the MeOH concentration on the photoenzymatic hydroxylation of ethylbenzene; reaction conditions: *Aae*UPO (25 nm), ethylbenzene (10 mM), CN-OA-m (2.0 mg/mL), MeOH (0-20% v/v), tricine buffer (100 mM, pH 7.5), 528 nm (1330 µmol photons m^{-2} s^{-1}), 30 °C, 24 h.

Influence of the Enzyme Concentration

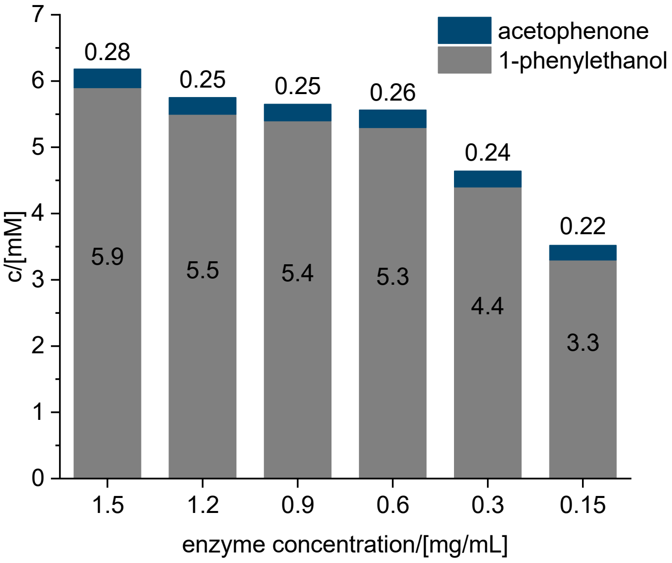

Figure S45. Influence of the enzyme concentration on the photoenzymatic hydroxylation of ethylbenzene; reaction conditions: *Aae*UPO (1.5-0.15 mg/mL), ethylbenzene (10 mM), CN-OA-m (2.0 mg/mL), MeOH (250 mM), tricine buffer (100 mM, pH 7.5), 528 nm (1330 µmol photons m^{-2} s^{-1}), 30 °C, 24 h.

Influence of the Amount of CN-OA-m

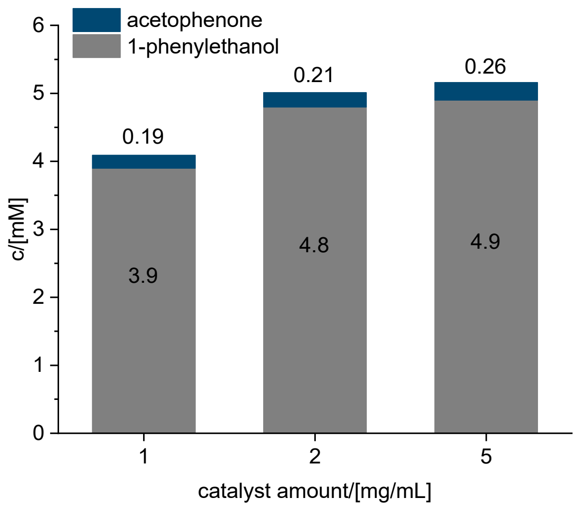

Figure S46. Influence of the CN-OA-m concentration on the photoenzymatic hydroxylation of ethylbenzene; reaction conditions: *Aae*UPO (25 nM), ethylbenzene (10 mM), CN-OA-m (1.0-5.0 g/mL), MeOH (250 mM), tricine buffer (100 mM, pH 7.5), 528 nm (1330 µmol photons m^{-2} s^{-1}), 30 °C, 24 h.

Influence of the Light Intensity

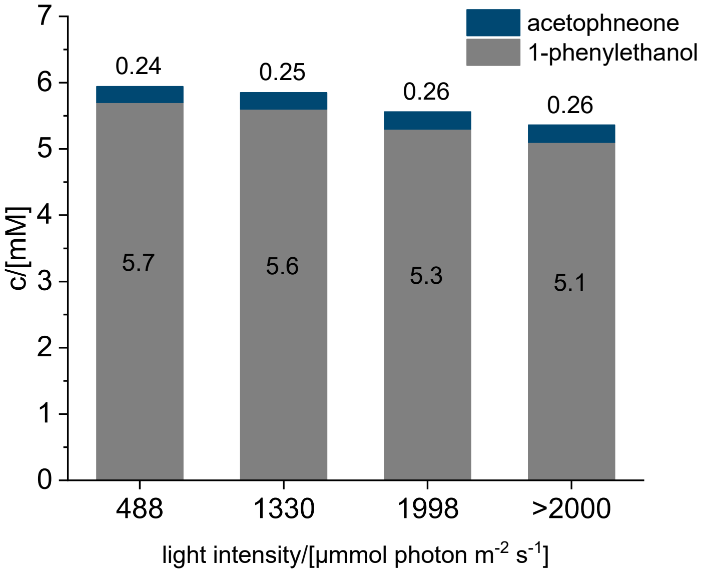

Figure S47. Influence of the light intensity on the photoenzymatic hydroxylation of ethylbenzene; reaction conditions: *Aae*UPO (25 nM), ethylbenzene (10 mM), CN-OA-m (2.0 mg/mL), MeOH (250 mM), tricine buffer (100 mM, pH 7.5), 528 nm (488->2000 µmol photons m^{-2} s^{-1}), 30 °C, 24 h.

*Aae*UPO Stability at Different Wavelengths

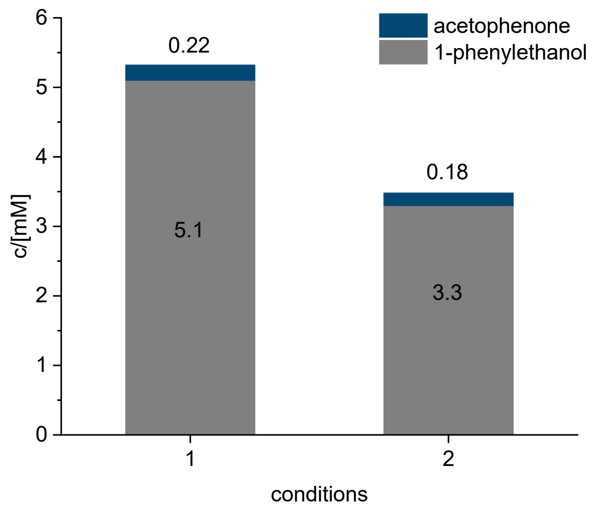

Figure S48. Influence of the wavelength on the photoenzymatic hydroxylation of ethylbenzene; 1) Incubation of *Aae*UPO in tricine buffer at 528 nm for 1 h (without substrate); 2) Incubation of *Aae*UPO in tricine buffer at 455 nm for 1 h (without substrate); After pre-incubation, ethylbenzene (10 mM) was added and the reactions were incubated at 528 nm for further 24 h; reaction conditions: *Aae*UPO (25 nM), ethylbenzene (10 mM), CN-OA-m (2.0 mg/mL), MeOH (250 mM), tricine buffer (100 mM, pH 7.5), 528 nm (1330 µmol photons m^{-2} s^{-1}), 30 °C, 24 h.

Reuse of CN-OA-m

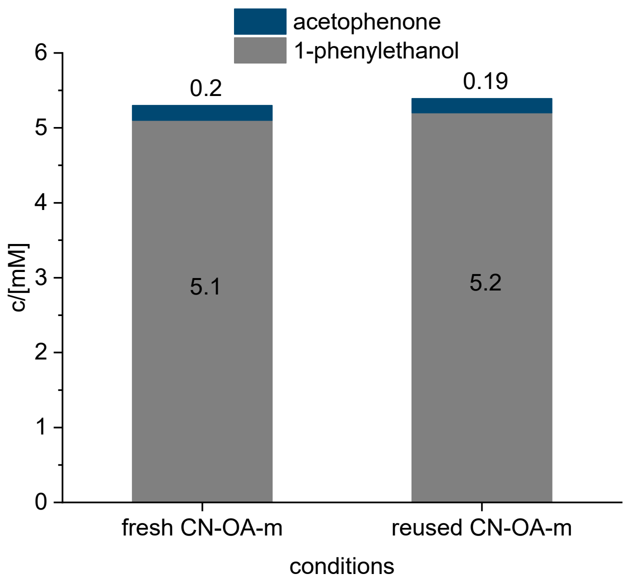

Figure S49. Influence of reused CN-OA-m on the photoenzymatic hydroxylation of ethylbenzene; reaction conditions: *Aae*UPO (25 nM), ethylbenzene (10 mM), CN-OA-m (2.0 mg/mL), MeOH (250 mM), tricine buffer (100 mM, pH 7.5), 528 nm (1330 µmol photons m^{-2} s^{-1}), 30 °C, 24 h.

Comparison of CN-OA-m before and after the Photo-Chemo-Enzymatic Reaction with AaeUPO

Transmission electron microscopy (TEM) was performed on a CM200F EG (Philips) microscope, operated at 200 kV.

Scanning electron microscopy (SEM) images were obtained on a LEO 1550-Gemini microscope.

Energy-dispersive X-ray (EDX) investigations were conducted on a Link ISIS-300 system (Oxford Microanalysis Group) equipped with a Si(Li) detector and an energy resolution of 133 eV.

Table S6. EDX elemental composition acquired from new and recovered CN-OA-m.

Sample	% w/w N	% w/w C	% w/w O	% w/w K	% w/w Fe
**CN-OA-m**	45.91	38.47	5.64	9.51	0.01
**CN-OA-m recovered**	57.34	30.97	7.94	1.91	0.03

The SEM, as well as the TEM images, show the same morphology and no altering during the catalytic transformation

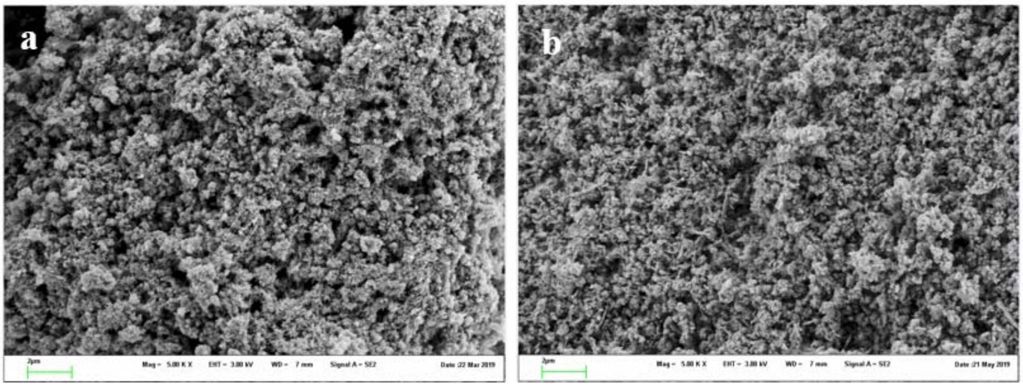

Figure S50. a) SEM images of CN-OA-m new; b) recovered CN-OA-m.

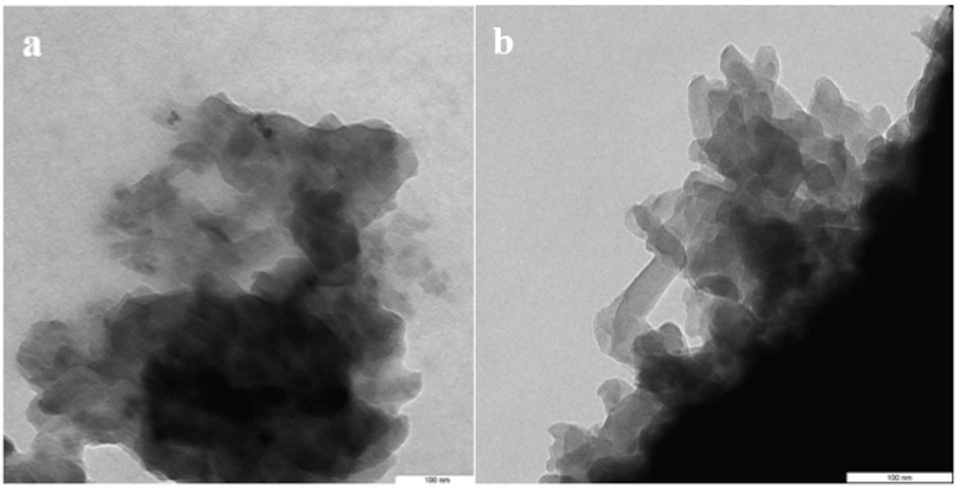

Figure S51. a) TEM images of CN-OA-m new; b) recovered CN-OA-m.

DNA and Protein Sequences

5`-
catatgGCCAGCTGGAGTCATCCGCAGTTTGAAAAAGGCGCCGAGTTTATGAAAGCAGTGCAGTATACC
GAAATTGGCAGTGAACCGGTTGTTGTTGATATTCCGACCCCGACCCCGGGCCCGGGTGAAATTCTGC
TGAAAGTGACCGCAGCAGGCCTGTGCCATAGTGATATTTTTGTGATGGATATGCCGGCAGCACAGTA
TGCATACGGTCTGCCGCTGACCCTGGGTCATGAAGGTGTGGGTACCGTTGCCGAACTGGGCGAAGG
TGTGACCGGTTTTGGTGTGGGTGACGCCGTTGCAGTTTATGGCCCGTGGGGCTGCGGTGCATGTCA
TGCATGCGCACGCGGCCGCGAAAATTATTGTACCCGTGCAGCAGATCTGGGTATTACCCCGCCGGG
CCTGGGCAGCCCTGGTAGCATGGCTGAATATATGATTGTTGATAGCGCCCGTCATCTGGTTCCGATT
GGCGATCTGGATCCGGTGGCCGCCGCCCCTCTGACAGATGCTGGTCTGACCCCGTATCATGCAATT
AGCCGTGTTCTGCCGCTGCTGGGTCCGGGTAGCACCGCAGTGGTTATTGGTGTTGGTGGCCTGGGT
CATGTTGGTATTCAGATTCTGCGTGCCGTGAGTGCCGCACGCGTGATTGCCGTGGATCTGGATGATG
ATCGTCTGGCCCTGGCCCGTGAAGTTGGTGCCGATGCAGCCGTTAAAAGTGGTGCAGGCGCCGCCG
ATGCAATTCGTGAACTGACCGGTGGTCAGGGCGCAACCGCCGTTTTTGATTTTGTTGGCGCACAGAG
CACCATTGATACCGCACAGCAGGTTGTTGCCGTGGATGGTCATATTAGCGTGGTTGGCATTCATGCA
GGCGCCCATGCCAAAGTTGGTTTCTTTATGATTCCGTTTGGTGCAAGCGTTGTGACCCCGTATTGGG
GTACCCGTAGCGAACTGATGGAAGTTGTGGCACTGGCACGTGCCGGTCGTCTGGATATTCATACCGA
AACCTTTACCCTGGATGAAGGCCCGGCCGCATATCGCCGCCTGCGTGAAGGTAGTATTCGCGGTCG
TGGCGTGGTTGTTCCGTAActcgag-3´

DNA sequence of ADH-A with strep-tag sequence and restriction sites (*Nde*I and *Xho*I).

HMASWSHPQFEKGAEFMKAVQYTEIGSEPVVVDIPTPTPGPGEILLKVTAAGLCHSDIFVMDMPAAQYAY
GLPLTLGHEGVGTVAELGEGVTGFGVGDAVAVYGPWGCGACHACARGRENYCTRAADLGITPPGLGSP
GSMAEYMIVDSARHLVPIGDLDPVAAAPLTDAGLTPYHAISRVLPLLGPGSTAVVIGVGGLGHVGIQILRAV
SAARVIAVDLDDDRLALAREVGADAAVKSGAGAADAIRELTGGQGATAVFDFVGAQSTIDTAQQVVAVDG
HISVVGIHAGAHAKVGFFMIPFGASVVTPYWGTRSELMEVVALARAGRLDIHTETFTLDEGPAAYRRLREG
SIRGRGVVVP-

Protein sequence of ADH-A and fused strep-tag (yellow).

Abbreviations

ADH	alcohol dehydrogenase
amp	ampicillin
CV	column volume
EtOH	ethanol
IPTG	isopropyl *β*-D-1-thiogalactopyranoside
*i*PrOH	isopropanol
kan	kanamycin
LED	light-emitting diode
MeOH	methanol
NAD^+	nicotinamide adenine dinucleotide
NADH	reduced nicotinamide adenine dinucleotided
$NADP^+$	nicotinamide adenine dinucleotide phosphate
NADPH	reduced nicotinamide adenine dinucleotide phosphate
NMR	nuclear magnetic resonance
OD_{600}	optical density at 600 nm
ONC	overnight culture
SDS-PAGE	sodium dodecyl sulfate polyacrylamide gel
UPO	unspecific peroxygenase

Literature

[1] a) B. Pieber, J. A. Malik, C. Cavedon, S. Gisbertz, A. Savateev, D. Cruz, T. Heil, G. Zhang, P. H. Seeberger, *Ange. Chem. Int. Ed.* **2019**, *58*, 9575-9580; *Angew. Chem.* 2019, 131, 9678-9681; b) G. Zhang, G. Li, Z.-A. Lan, L. Lin, A. Savateev, T. Heil, S. Zafeiratos, X. Wang, M. Antonietti, *Angew. Chem. Int. Ed.* **2017**, *56*, 13445-13449; *Angew. Chem.* **2017**, *129*, 13630-13634.

[2] G. R. Fulmer, A. J. M. Miller, N. H. Sherden, H. E. Gottlieb, A. Nudelman, B. M. Stoltz, J. E. Bercaw, K. I. Goldberg, *Organometallics* **2010**, *29*, 2176-2179.

[3] H. E. Bonfield, J. D. Williams, W. X. Ooi, S. G. Leach, W. J. Kerr, L. J. Edwards, *ChemPhotoChem* **2018**, *2*, 938-944; bH. E. Bonfield, K. Mercer, A. Diaz-Rodriguez, G. C. Cook, B. S. J. McKay, P. Slade, G. M. Taylor, W. X. Ooi, J. D. Williams, J. P. M. Roberts, J. A. Murphy, L. Schmermund, W. Kroutil, T. Mielke, J. Cartwright, G. Grogan, L. J. Edwards, *ChemPhotoChem* **2020**, *4*, 45-51.

[4] J. Xiao, Z. Z. Wong, Y. P. Lu, T. P. Loh, *Adv. Synth. Catal.* **2010**, *352*, 1107-1112.

[5] D.-M. Du, T. Fang, J. Xu, S.-W. Zhang, *Org. Lett.* **2006**, *8*, 1327-1330.

[6] H. L. Holland, E. J. Bergen, P. C. Chenchaiah, S. H. Khan, B. Munoz, R. W. Ninniss, D. Richards, *Can. J. Chem.* **1987**, *65*, 502-507.

[7] a) M. Hatano, T. Miyamoto, K. Ishihara, *J. Org. Chem.* **2006**, *71*, 6474-6484; b) B. Zhang, H. Wang, G.-Q. Lin, M.-H. Xu, *Eur. J. Org. Chem.* **2011**, *2011*, 4205-4211.

[8] S. A. Moteki, J. M. Takacs, *Angew. Chem. Int. Ed.* **2008**, *47*, 894-897; *Angew. Chem.* **2008**, *120*, 908-911.

[9] M. Hatano, O. Ito, S. Suzuki, K. Ishihara, *J. Org. Chem.* **2010**, *75*, 5008-5016.

[10] S. Yamada, T. Misono, Y. Iwai, A. Masumizu, Y. Akiyama, *J. Org. Chem.* **2006**, *71*, 6872-6880.

[11] E. Fernández-Mateos, B. Maciá, M. Yus, *Tetrahedron: Asymmetry* **2012**, *23*, 789-794.

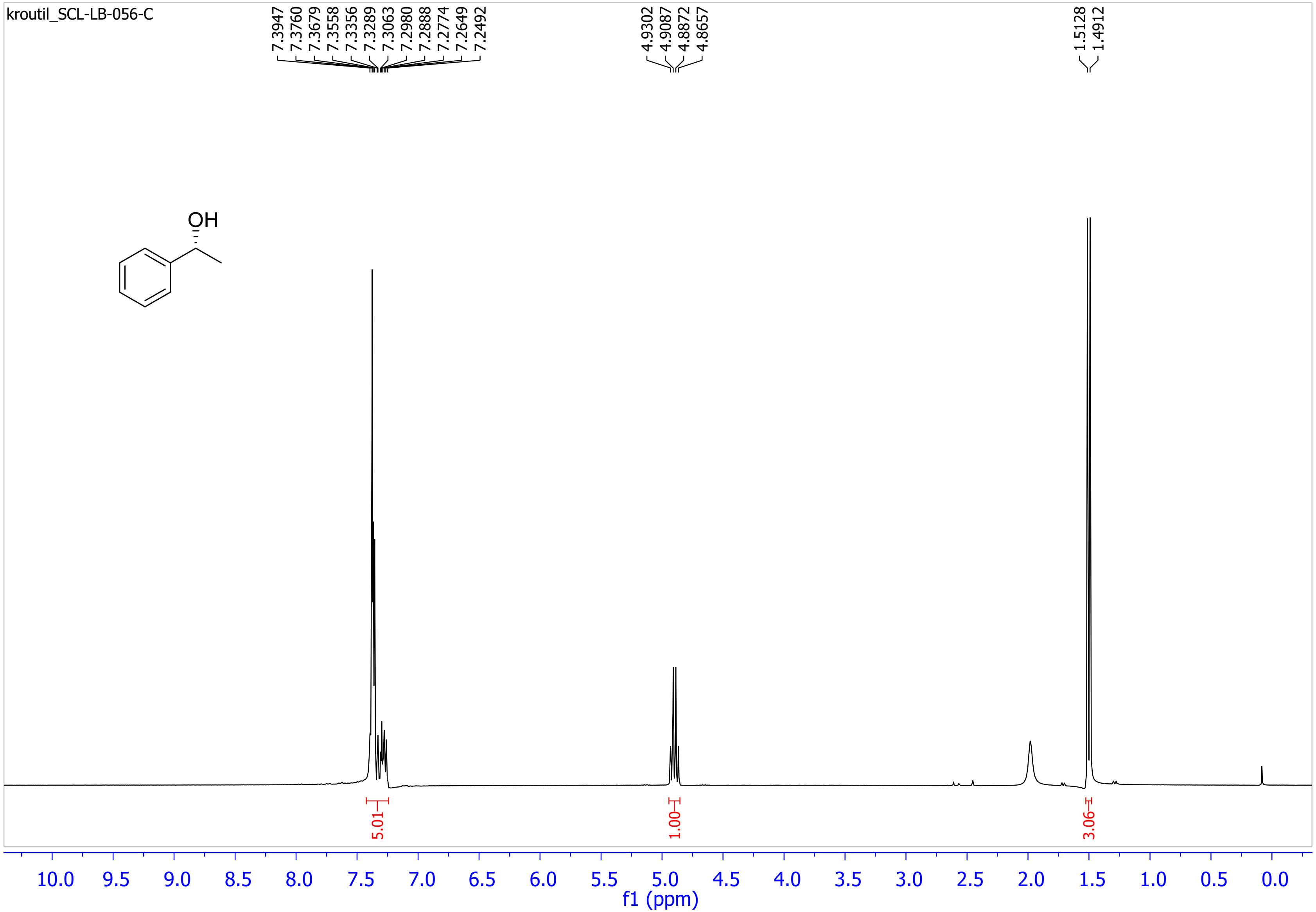

Figure S52. ^{1}H-NMR of (*R*)-1-phenylethanol.

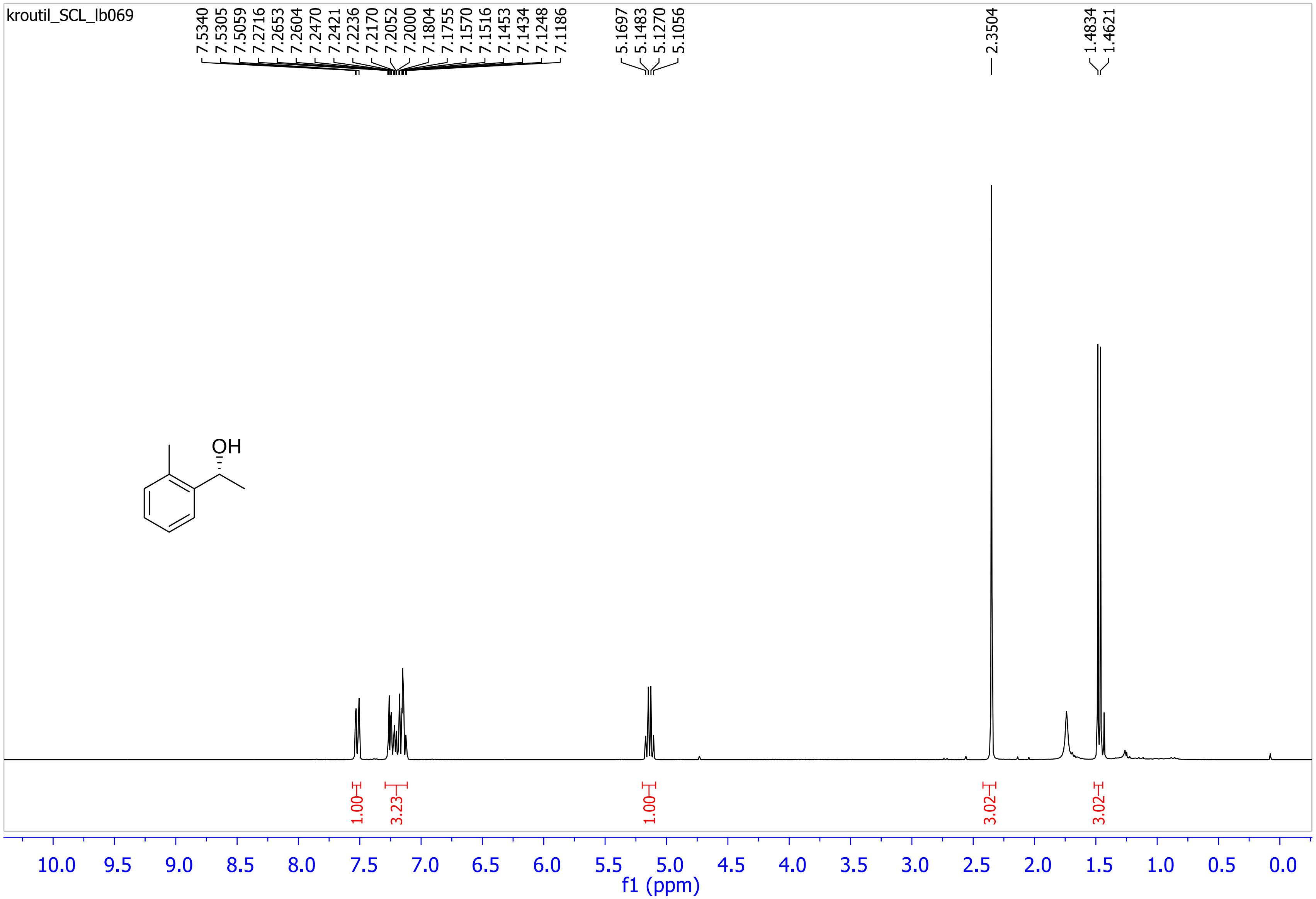

Figure S53. ^{1}H-NMR of (*R*)-1-(*o*-tolyl)ethan-1-ol.

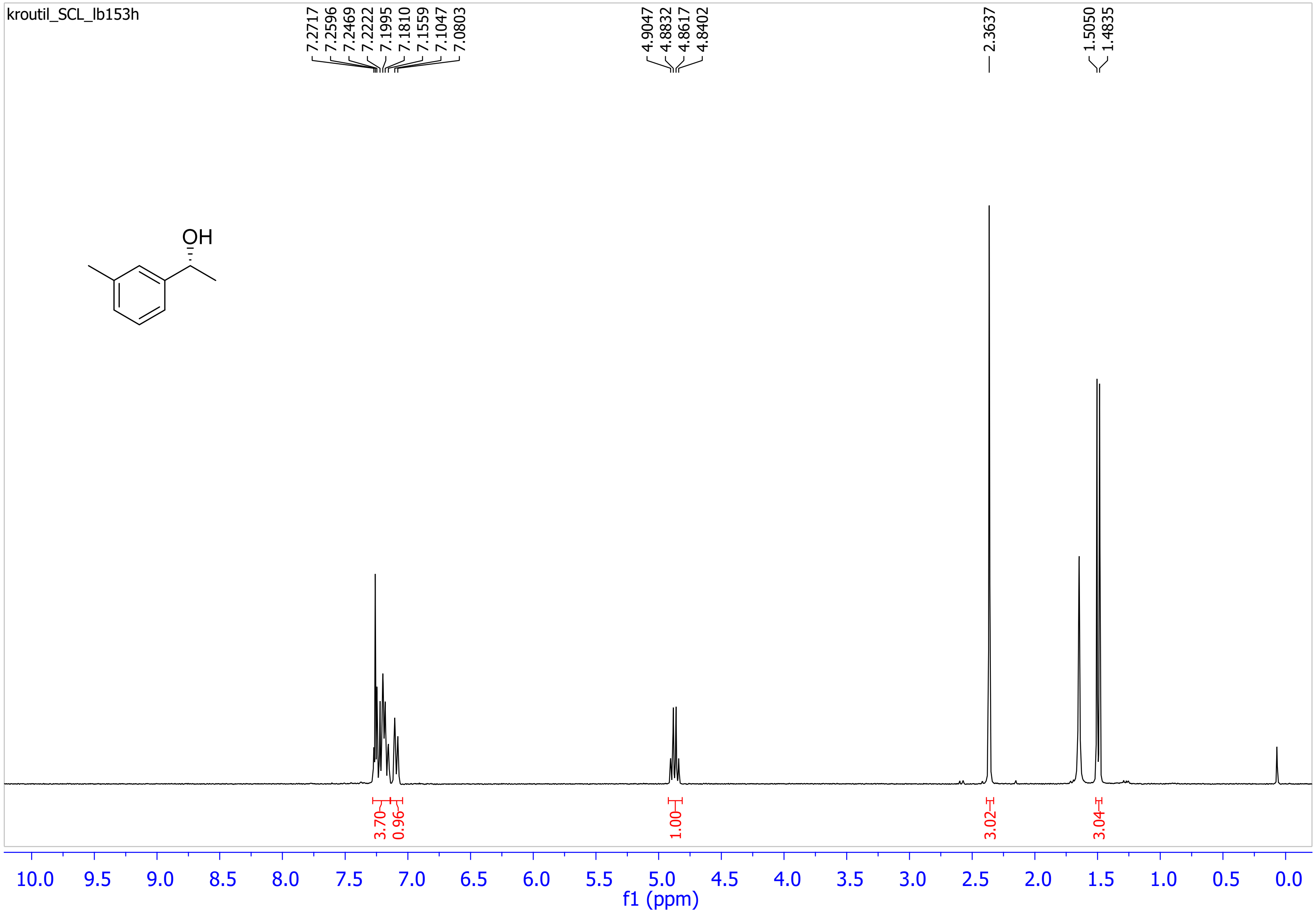

Figure S54. ^{1}H-NMR of (*R*)-1-(*m*-tolyl)ethan-1-ol.

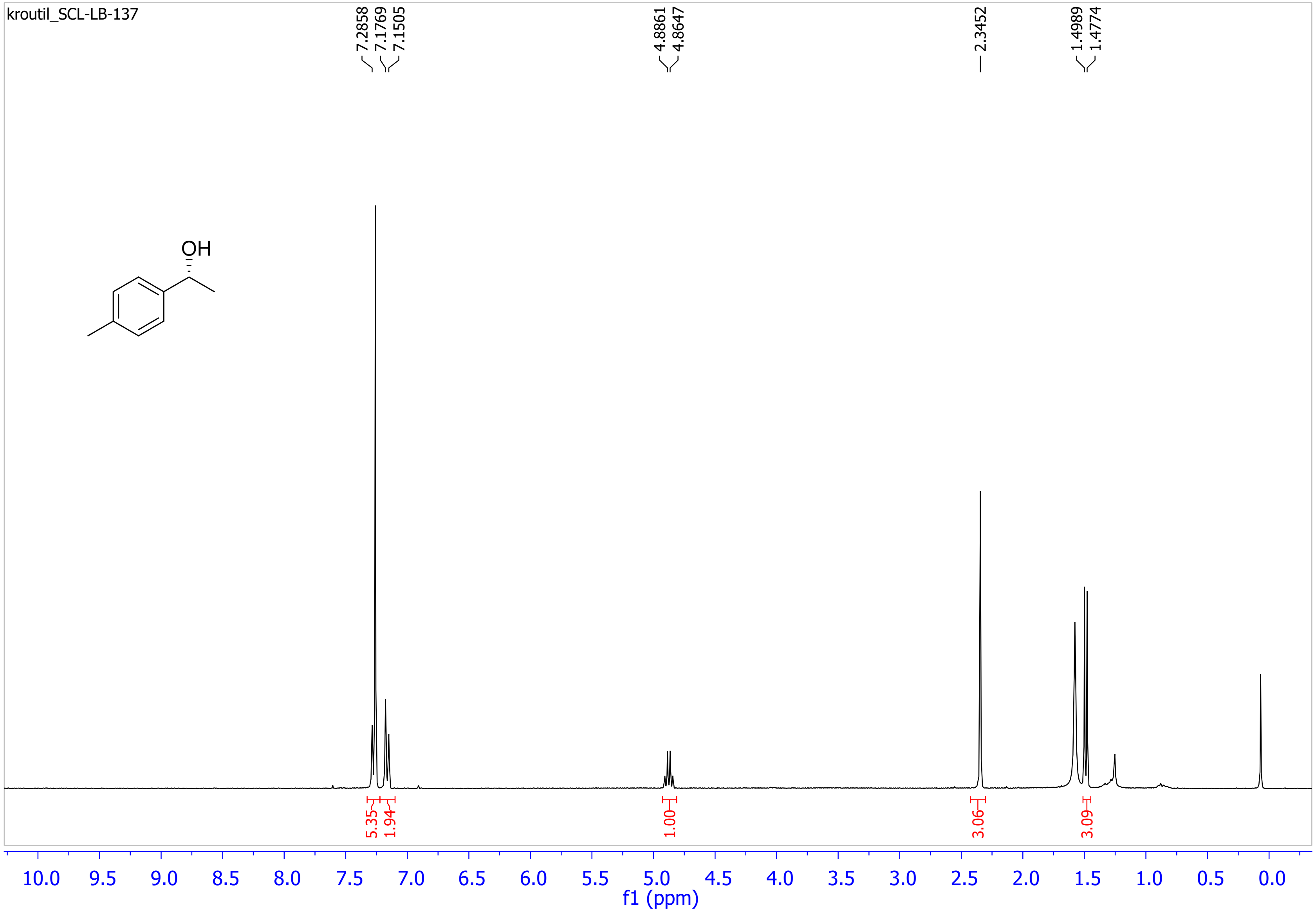

Figure S55. ^{1}H-NMR of (*R*)-1-(*p*-tolyl)ethan-1-ol.

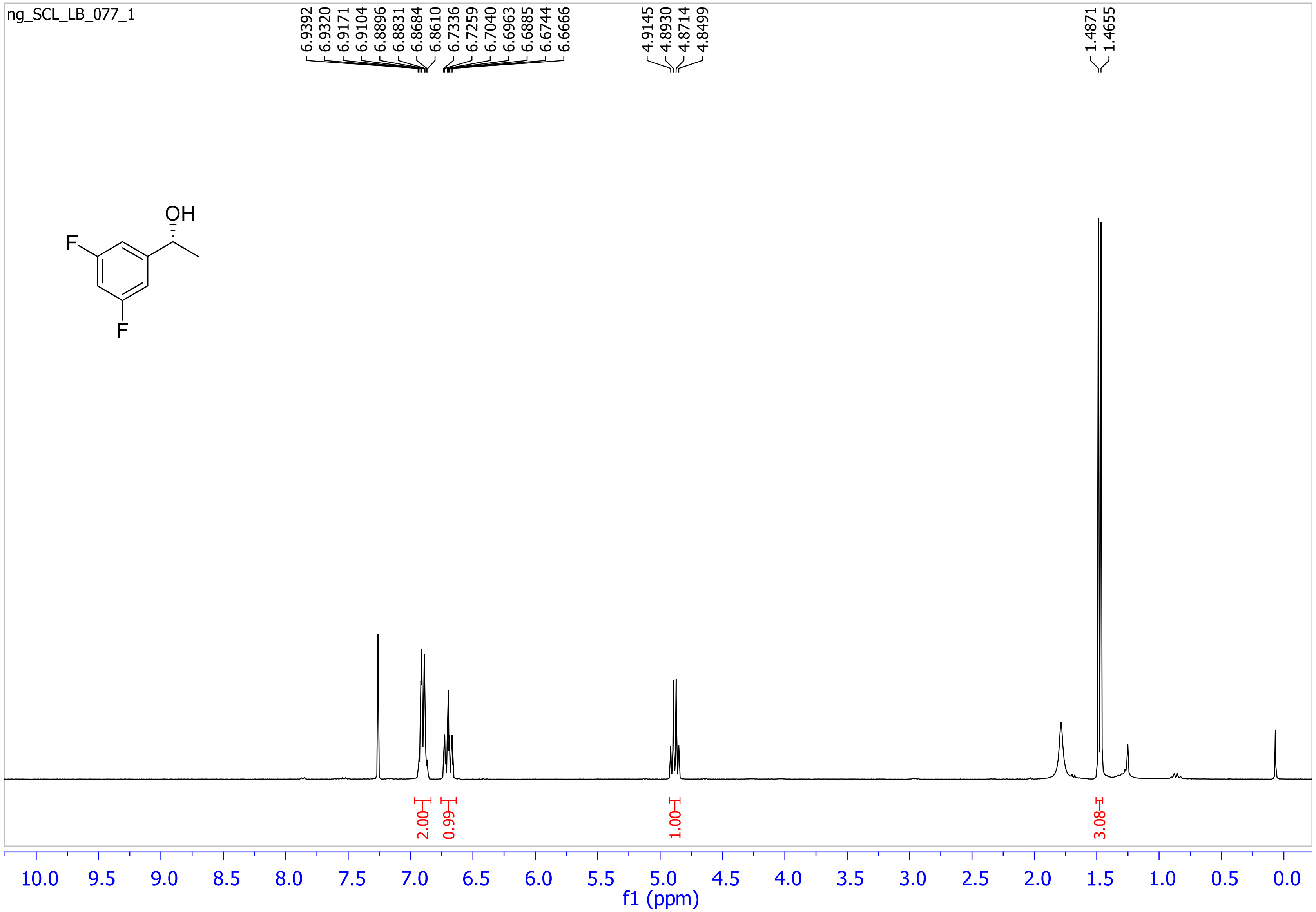

Figure S56. ^{1}H-NMR of (*R*)-1-(3,5-difluorophenyl)ethan-1-ol.

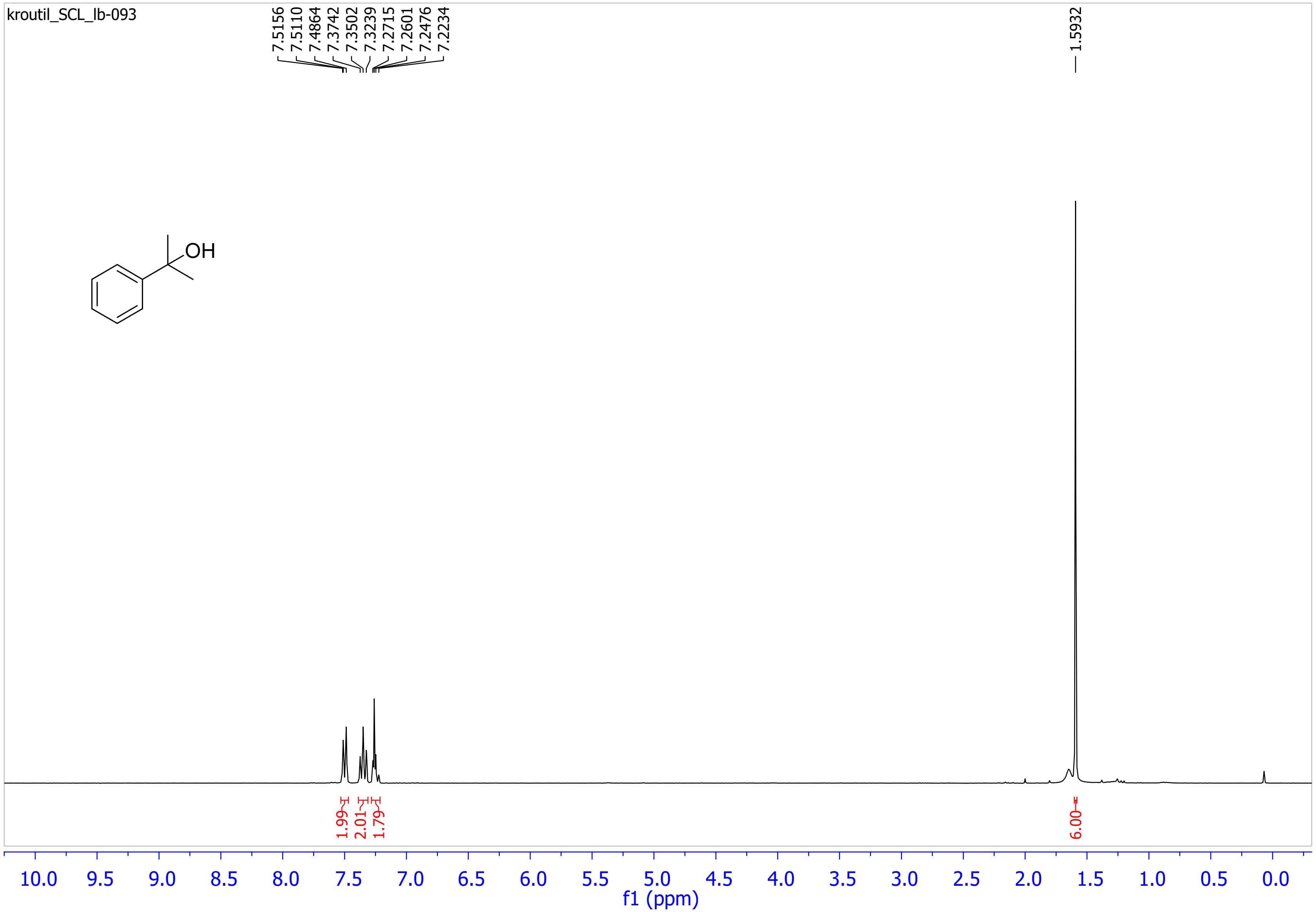

Figure S57. ^{1}H-NMR of 2-phenylpropan-2-ol.

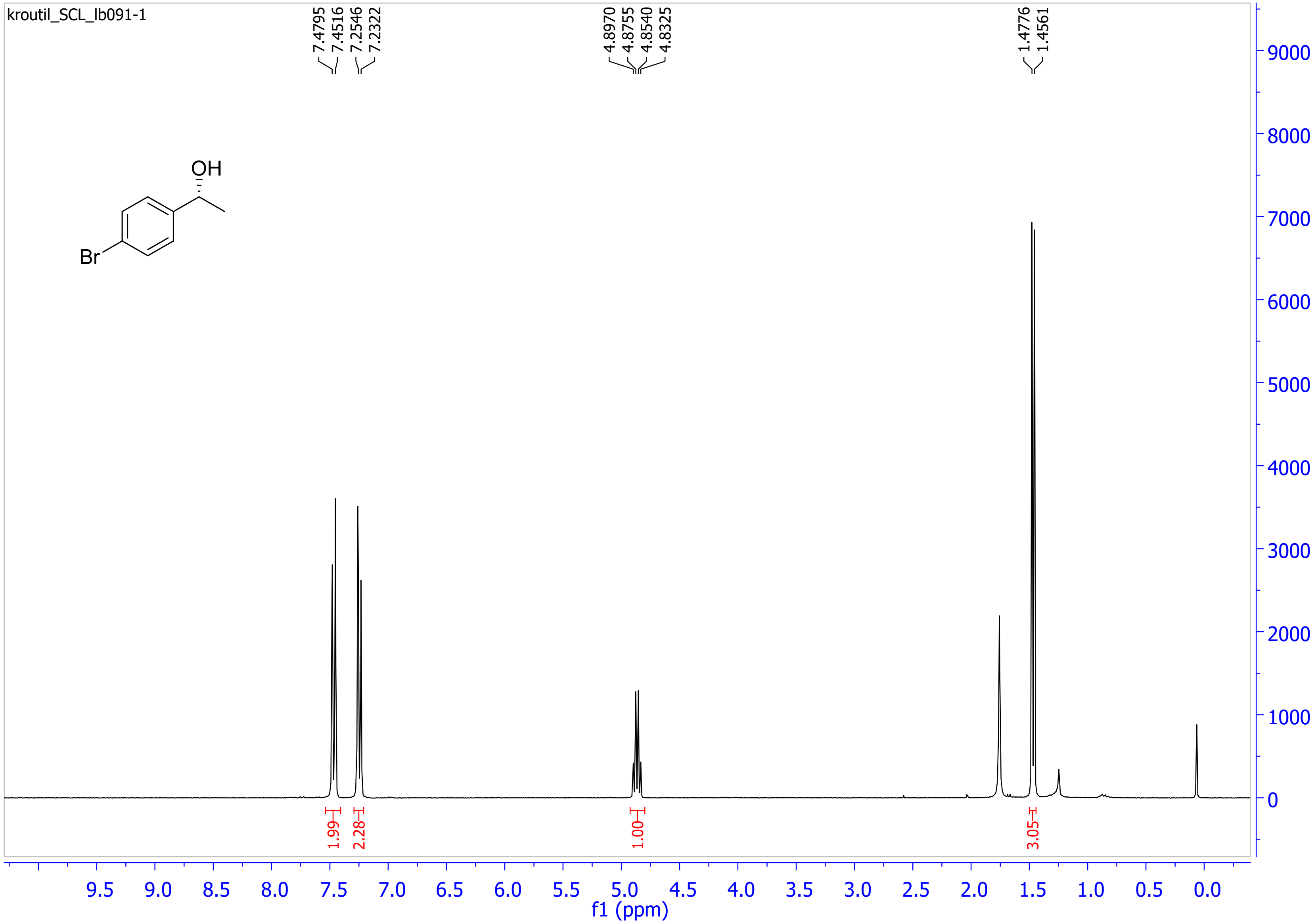

Figure S58. ^{1}H-NMR of (*R*)-1-(4-bromophenyl)ethan-1-ol.

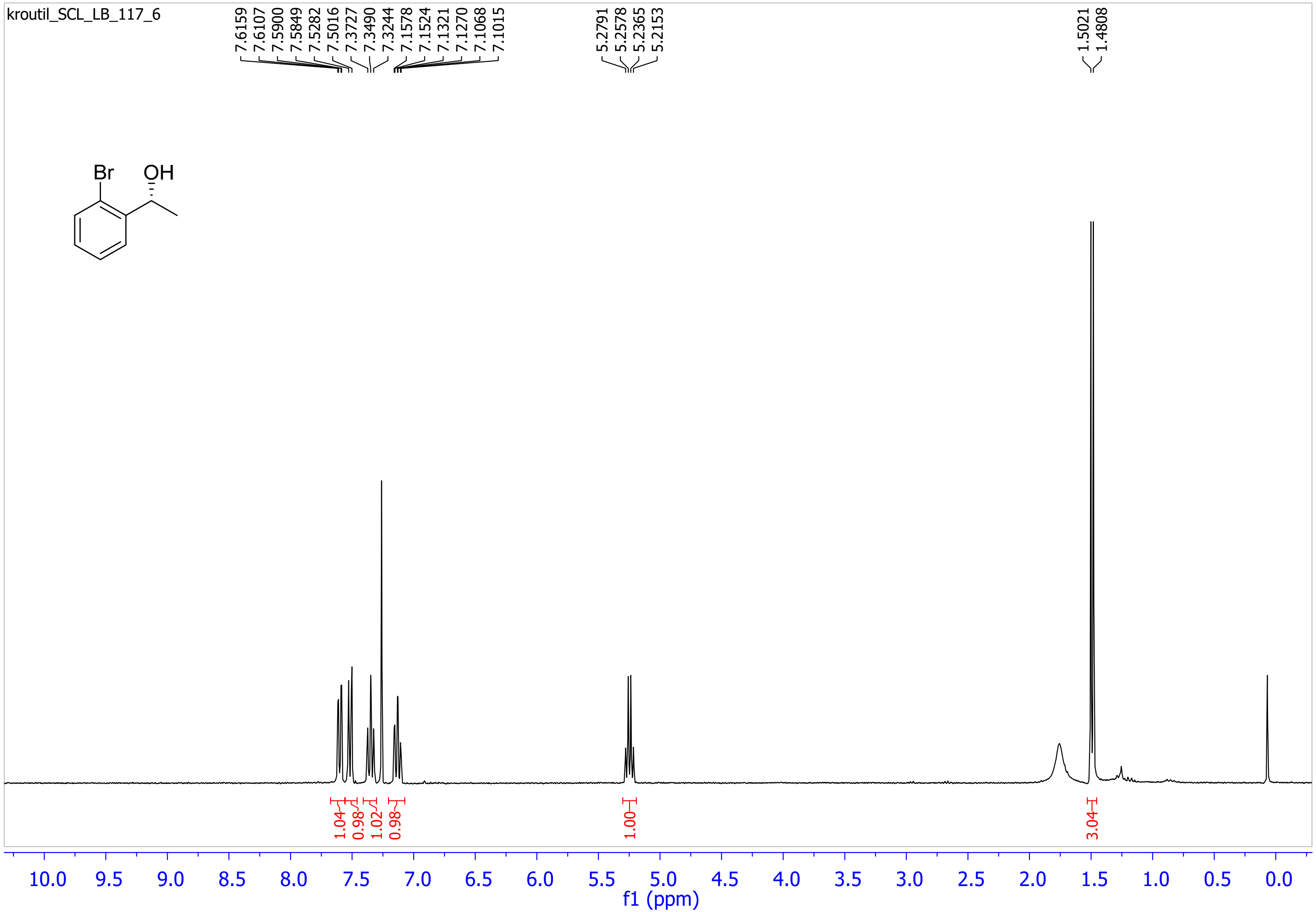

Figure S59. ^{1}H-NMR of (*R*)-1-(2-bromophenyl)ethan-1-ol.

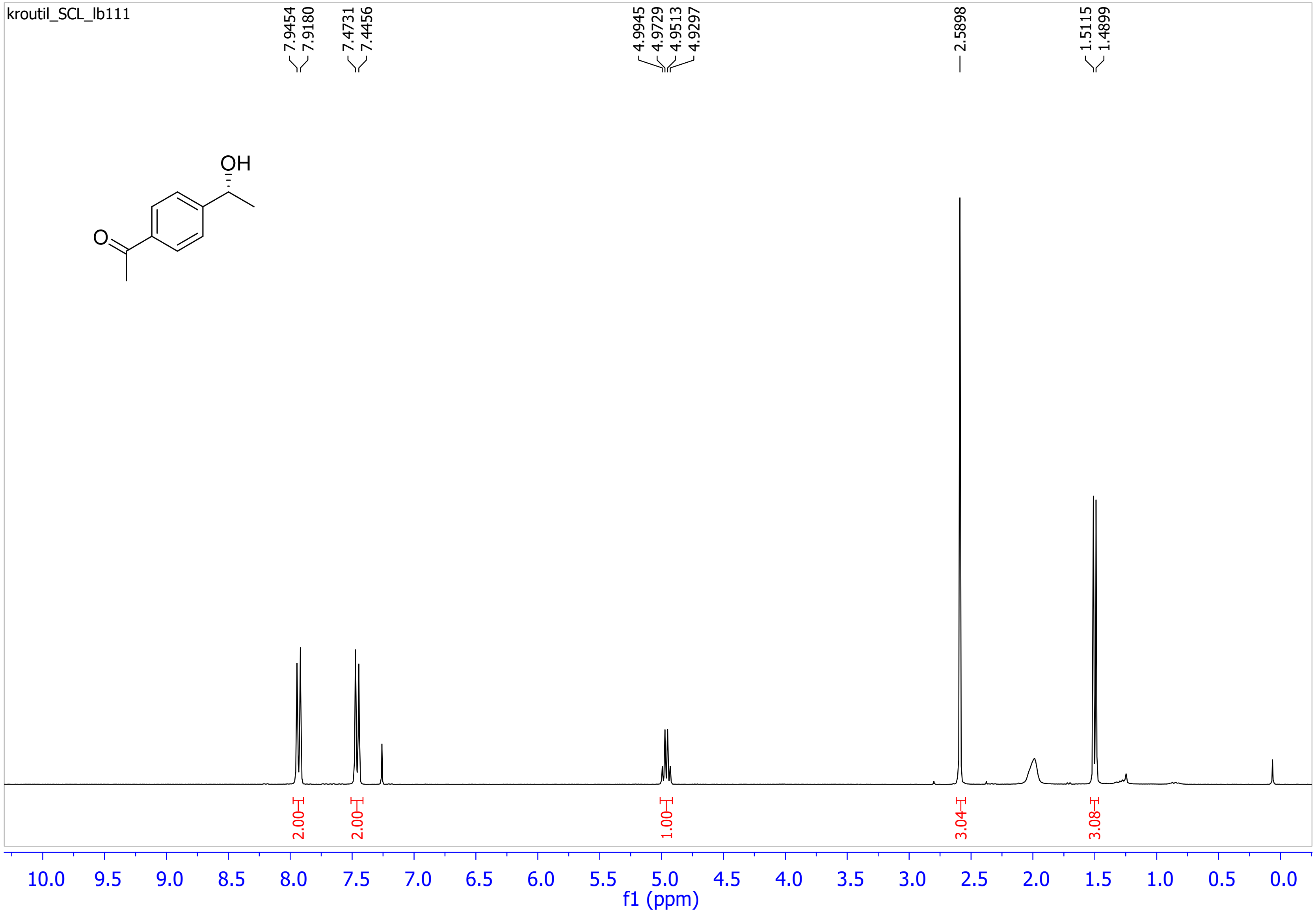

Figure S60. ^{1}H-NMR of (*R*)-1-(4-(1-hydroxyethyl)phenyl)ethan-1-one.

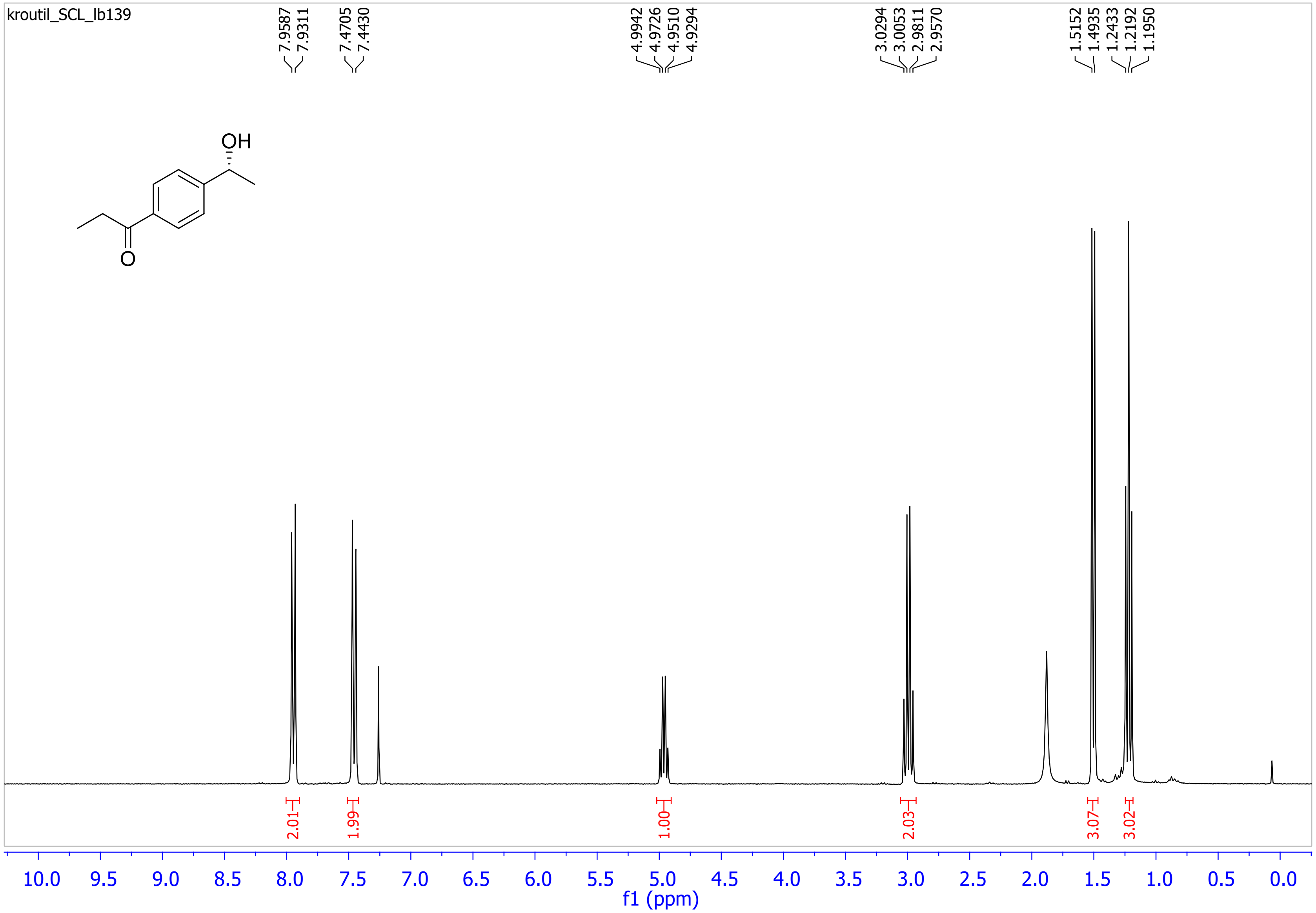

Figure S61. ^{1}H-NMR of (*R*)-1-(4-(1-hydroxyethyl)phenyl)propan-1-one.
